# The Obstacles and Potential Solution Clues of Prime Editing Applications in Tomato

**DOI:** 10.34133/bdr.0001

**Published:** 2022-12-15

**Authors:** Tien Van Vu, Ngan Thi Nguyen, Jihae Kim, Swati Das, Jinsu Lee, Jae-Yean Kim

**Affiliations:** ^1^ Division of Applied Life Science (BK21 FOUR Program), Plant Molecular Biology and Biotechnology Research Center, Gyeongsang National University, Jinju 660-701, Republic of Korea.; ^2^ National Key Laboratory for Plant Cell Biotechnology, Agricultural Genetics Institute, Km 02, Pham Van Dong Road, Co Nhue 1, Bac Tu Liem, Hanoi 11917, Vietnam.; ^3^ Division of Life Science, Gyeongsang National University, 501 Jinju-daero, Jinju 52828, Republic of Korea.; ^4^ Nulla Bio Inc., 501 Jinju-daero, Jinju 660-701, Republic of Korea.

## Abstract

Precision genome editing is highly desired for crop improvement. The recently emerged CRISPR/Cas technology offers great potential applications in precision plant genome engineering. A prime editing (PE) approach combining a reverse transcriptase (RT) with a Cas9 nickase and a “priming” extended guide RNA (gRNA) has shown a high frequency for precise genome modification in mammalian cells and several plant species. Nevertheless, the applications of the PE approach in dicot plants are still limited and inefficient. We designed and tested prime editors for precision editing of a synthetic sequence in a transient assay and for desirable alleles of 10 loci in tomato by stable transformation. Our data obtained by targeted deep sequencing also revealed only low PE efficiencies in both the tobacco and tomato systems. Further assessment of the activities of the PE components uncovered that the fusion of RT to Cas9 and the structure of PE gRNAs (pegRNAs) negatively affected the cleaving activity of the Cas9 nuclease. The self-complementarity between the primer binding sequences (PBSs) and spacer sequence might pose risks to the activity of the Cas9 complex. However, modifying the pegRNA sequences by shortening or introducing mismatches to the PBSs to reduce their melting temperatures did not enhance the PE efficiency at the MADS-box protein (SlMBP21), alcobaca (SlALC), and acetolactate synthase 1 (SlALS1) loci. Our data show challenges of the PE approach in tomato, indicating that a further improvement of the PE system for successful applications is demanded, such as the use of improved expression systems for enriching active PE complexes.

## Introduction

RNAs have been used for gene correction in human and yeast cells [1]. CRISPR/Cas-mediated precision editing using a chimeric single-guide RNA (sgRNA) and a donor template was shown in rice protoplasts [2,3]. However, the gene targeting (GT) frequency recorded using the RNA donor was much lower than that of a single-stranded DNA donor [3]. Further work needs to be done on using RNA as a template for precision plant gene editing.

Recently, prime editing (PE) using gRNA extensions for priming reverse transcription-mediated precise editing appeared to be an excellent precision genome editing technique in mammalian cell lines [4]. The best version of the prime editor used a CRISPR/Cas complex developed by fusing a reverse transcriptase (RT) to the C-terminus of a Cas9 nickase (H840A) and a PE gRNA (pegRNA) with a 3′ extension that could bind to the 3′ nicked strands produced by nCas9. When bound, the nicked strand’s free 3′-OH is used as the substrate for the RT to copy genetic information from the pegRNA’s 3′ extension. If pegRNAs were designed to produce modified nucleotides, then these nucleotides would be inserted into the genome during downstream repair processes [4]. A second nick site present downstream of the first nick site would support the retention of the introduced nucleotides. The PE approach may also be an excellent alternative to GT with shorter editing sequence coverage [5]. Prime editors have been shown to work well in monocot plants by Lin and coworkers [6]. In the same year, other reports also showed the activities of PE complexes in monocots such as rice [7–11] and maize [12]. However, most of the data showed successful PE at the acetolactate synthase (ALS) locus. Minimal data regarding the applications of PE in dicots were released, with relatively low efficacy in tobacco and *Arabidopsis* [13], potatoes [14], and tomato [15], and this process would need to be improved for further applications.

Therefore, we sought to investigate the activity of PE complexes in plants using a transient assay with a synthetic substrate in tobacco and stable transformation for editing 10 loci in tomatoes. Our data showed low PE efficiency at both the somatic cell and plant levels. Additional analyses revealed the possible adverse effects of the PE components on PE performance. The Cas9–RT fusion and the self-complementary structure of pegRNAs negatively affected the cleaving activity of the Cas9 nuclease. We further modified the primer binding sequences (PBSs) by shortening or introducing mismatches but did not significantly improve the PE efficiency. Our work shows obstacles and essential clues for further improving the PE approach in plants.

## Materials and Methods

### System design for PE experiments in tobacco and tomato

For the transient experiment, PE constructs were designed to modify 2 nucleotides of the (CA)n substrate sequence using the 3′ extension pegRNA (Fig. S1A and B and Data S1). A tobacco codon-optimized RT DNA sequence was fused with a human codon-optimized SpCas9 nickase (H840A) (nCas9–RT; Data S1) cloned using SpCas9 of the Addgene plasmid (#49771) that worked well in plants [16]. The PE experiment was performed by using dual agroinfiltration (Fig. S1C). For comparison, cytidine base editor (CBE) constructs were designed to convert C’s to T’s, including the PE-targeted C at position 17 counted from the Protospacer Adjacent Motif (PAM) of the CBE gRNA (Fig. S1B).

**Fig. 1.**
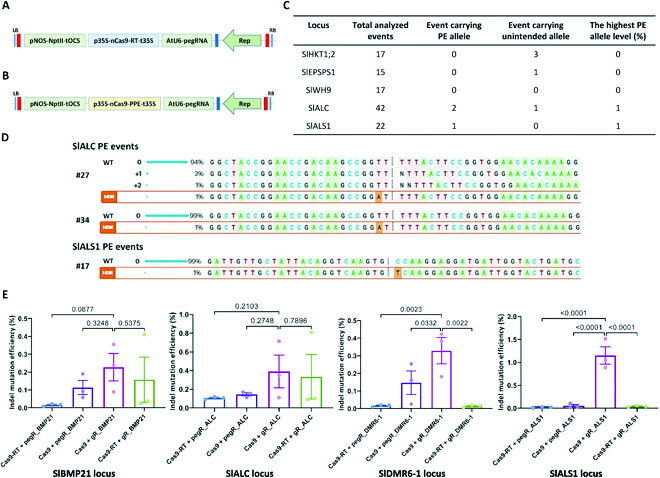
Prime editing (PE) performance in tomato. (A and B) PE2 PE constructs using the geminiviral replicon in tomato with synthetic tobacco codon-optimized RT (A) (7 loci) or rice codon-optimized PPE (B) (10 loci). The NptII expression cassette (Addgene, plasmid #51144) was used for the kanamycin selection of transformed cells, tissues, and plants. The expression of the prime editors was driven by the p35S promoter with the UBQ10 intron 1 and CaMV 35S terminator (Data S1). The transcription of the pegRNAs was controlled by the AtU6 core promoter sequence (Data S1). (C) PE performance data obtained from the analysis of the plant–stage transformants of SlWH9, SlALC, and SlALS1 by Sanger sequencing and Inference of CRISPR Edits (ICE) Synthego. (D) The traces of PE-edited alleles were obtained for SlALC (upper 2 panels) and SlALS1 (bottom panel). The precisely edited alleles are shown at the bottom of each panel and are denoted by “HDR” labels. The PE-mediated replaced bases are highlighted with an orange background. (E) Scatter dot–bar plots showing the pairwise comparisons between the indel mutation efficiencies of the Cas9–gRNA complexes with the PE components and that of the normal construction at the SlMBP21, SlALC, SlDMR6-1, and SlALS1. The comparison at each locus is presented with the locus name at the bottom of the plot. The combinations (denoted as “+”) of nuclease complexes are shown at the bottom of each scattered bar with Cas proteins (Cas9 and Cas9–RT fusion) and gRNAs (normal gRNA with gR prefix and pegRNA with pegR prefix, followed by the locus name). The sequences flanking the targeted sites were amplified at 10 dpt and analyzed by targeted deep sequencing in three replicates. The indel mutation efficiencies were revealed by the RGEN Cas-Analyzer. Multiple comparisons of the means and plotting were conducted by GraphPad Prism version 9 using uncorrected Fisher’s LSD test. LB and RB in (A) and (B) denote left and right border, respectively. WT in (D) represents the wild-type allele at the loci. The *P* values of each compared mean pair are shown on the top of the bars.

For PE of the selected tomato loci using *Agrobacterium*-mediated stable transformation (Fig. S2), PE2 and PE3b approaches were applied for high-affinity K+ transporter 1;2 (SlHKT1;2), 5-Enolpyruvylshikimate-3-phosphate synthase 1 (SlEPSPS1), and orange (SlOr) (Figs. S3 and S4). Further applications of the nCas9–RT (tobacco codon-optimized RT (PE2) [4]), nCas9-prime editor system (PPE) (rice codon-optimized RT [6]) systems using the original or modified PBSs on the other 7 loci (Table S3) used a PE2 strategy (Fig. 1A and B and Data S1).

In all binary vectors, the transcription of both CBE and nCas9–RT was driven by a long Cauliflower Mosaic Virus 35S (CaMV35S) inserted with a polyubiquitin 10 (UBQ10) intron 1 [17]. The CBE gRNA and PE pegRNA were cloned downstream of an AtU6 promoter [16]. The CBE and PE systems were delivered by either transfer DNAs (T-DNAs) or geminiviral replicons [17].

### *Agrobacterium*-mediated infiltration and sample analysis

Leaves of tobacco (*Nicotiana benthamiana*) at the 6-leaf stage were infiltrated with *Agrobacteria* containing binary plasmids. *Agrobacteria* (GV3101::pMP90 strain) were prepared by an overnight primary culture with a subsequent 6- to 8-h secondary culture using liquid Luria-Bertani (LB) medium containing 75 mg/l of kanamycin, 30 mg/l of rifampicin, and 30 mg/l of streptomycin. *Agrobacterium* cells were collected by centrifugation at 4,000 revolutions/min for 10 min, resuspended in Murashige and Skoog (MS) buffer containing 100 μM acetosyringone to an OD_600nm_ (optical density at 600 nm) of 0.8, and incubated for 1 h before infiltration. At 3 d postinfiltration, the infiltrated zones were cut, and total genomic DNA was isolated using the Cetyltrimethyl Ammonium Bromide (CTAB) method. Polymerase chain reactions (PCRs) were then conducted using primers flanking the targeted sequences (Table S1), and the genomic DNA templates and PCR products were purified and sequenced by the Sanger method. The editing efficiency was calculated on the basis of the level of peaks of C and T at the targeted positions revealed by the chromatograms (Fig. S1D, E, G, and H).

### *Agrobacterium*-mediated tomato transformation and assessment of PE efficiency

*Agrobacterium*-mediated tomato transformation was performed using our in-house protocol (Fig. S2) [17]. Briefly, 7-day-old cotyledons were cut and used for transformation. For targeted deep sequencing, thin cotyledon explants (0.1 × 0.3 cm) were used to reduce the area containing untransformed cells beyond the cut edges of the explants. The *Agrobacteria* containing the PE constructs were cultured and harvested as in the transient assay but were resuspended in an Agrobacterium Minimal Medium (ABM)-MS solution [17] containing 100 μM acetosyringone to an OD_600nm_ of 0.8. The *Agrobacteria* were then incubated for 1 h before the transformation. Regenerated shoots were selected on a medium containing 80 mg/l of kanamycin. Subsequently, hardened plants were checked for PE activity.

For the assessment of PE efficiency, samples were collected at 10 d posttransformation (dpt) and analyzed by targeted deep sequencing. At the plant stage, plant leaves were collected and screened for PE alleles by Sanger sequencing, and targeted deep sequencing analysis was conducted for the events carrying PE alleles.

### Targeted deep sequencing

Genomic DNA was isolated from cotyledon explants or plant leaves using the CTAB method. The MiniSeq sequencing service (MiniSeq System, Illumina, USA) was used. MiniSeq samples were prepared in 3 PCRs according to the manufacturer’s guidelines, with genomic DNAs as templates for the first PCR. The first and second PCRs used the primers listed in Table S1, whereas the third PCR was conducted with the manufacturer's primers to assign sample IDs. The MiniSeq raw data FASTQ files were analyzed by the Cas-Analyzer tool [18] and PE-Analyzer (http://www.rgenome.net/pe-analyzer). The PE efficiency was assessed using the RT template sequences as the input homology-directed repair (HDR) donor sequence.

### Data analysis and presentation

Most of the experiments analyzed by targeted deep sequencing were conducted in 2 to 3 replicates. The editing data, statistical analysis if applicable, and scattered plots were further processed by the Microsoft Excel and GraphPad Prism 9.0 program. The multiple comparisons were conducted with the uncorrected Fisher’s least significant difference (LSD) test. The data are appropriately explained in detail in the legends of figures and/or tables.

## Results and Discussion

### The PE complexes did not perform better than the BE tool in a transient assay

The PE tool includes an nCas9 fused with an RT enzyme and a pegRNA for binding to the targeted sequence. Its 3′ extension primes an RNA-dependent DNA polymerization reaction by annealing to the upstream sequence of the nicked site on the nontargeted strand (Fig. S1A). If a second nick is simultaneously added, then we have a PE3 or PE3b approach, depending on the position of the second nick outside or inside the area covered by the RT, respectively [4]. To quickly validate the PE activity in plants, we started our PE experiments with a transient *Agrobacterium*-mediated infiltration system in tobacco leaves that was designed to modify a single nucleotide of a synthetic substrate ((CA)n substrate) (Fig. S1B and C). We reasoned that the nearly nondividing cells of a tobacco leaf might compromise PE efficiency since the active installation of modified bases by PE requires actively replicating cells and DNA repair system, and hence, targeting an endogenous gene of tobacco in the infiltration experiment might be less efficient. Recently, we have developed a geminiviral replicon system [17] that may facilitate the replication of the synthetic DNA substrate within a plant cell, thereby fulfilling the requirement of PE. Therefore, we hypothesized that the transient experiment using the replicon and synthetic substrate in tobacco leaves would quickly show the activities of the PE tools. A CBE system using PmCDA1 cytosine deaminase was used for comparison of the editing efficiency. The CBE design included an “-sgRNA” control (pCEC1) and 2 test vectors, 1 with the T-DNA (pCE01) and 1 with a geminiviral replicon system (pCE02) [17], for amplifying the editing tools (Fig. S1C). The PE3b tools were not only designed with both T-DNA and replicon cargos but also included dual vector systems to avoid possible activity in bacterial cells. The leaky expression of the CRISPR/Cas complexes in bacterial cells may lead to pre-editing the synthetic target during the cloning process, thereby complicating the data analysis in the plant. One vector carried the nCas9–RT expression cassette, the synthetic substrate, and a sgRNA expression cassette for generating a second nick (pPEsubc1 and pPEsubc2), and the other vector contained the pegRNA expression cassette (pPEsub1 and pPEsub2) (Fig. S1B and C). RT DNA was chemically synthesized as a tobacco codon-optimized Moloney murine leukemia virus RT based on the PE2 approach used in the work of Anzalone and coworkers [4]. pPEsubc1 and pPEsubc2 were also used alone as controls for the PE experiments. Three temperature conditions were applied to the infiltrated plants during the incubation stage to study the impacts of temperature on the activities of the BE and PE complexes. Surprisingly, we observed a very strong C to T transition at the 19th nucleotide counted from the PAM by BE in *Agrobacterium* cells (Fig. S1D and E, *Agrobacteria* panel) that led to indistinguishable BE activity at that position in tobacco leaves (Fig. S1D and E). By contrast, we observed BE activities in converting C to T at the 15th and 17th nucleotides in the tobacco leaves (Fig. S1D and E). Interestingly, the BE activities were reduced when the temperature increased from 25 to 37 °C (Fig. S1E and F and Table S2). Our data also indicate that the replicon-based BE tool worked much more efficiently than the T-DNA tool under transient expression conditions (Fig. S1E and F and Table S2). Unexpectedly, we failed to show any PE activity with the transient system using both the T-DNA and replicon systems. Furthermore, no improvement was obtained using the temperature treatments (Fig. S1G and H). One of the reasons for the failure of the PE3b constructs might be due to the inefficiency of the dual *Agrobacterium*-mediated infiltration that required simultaneous transfer of the 2 T-DNAs carrying PE components into 1 cell.

### The PE tools showed low-precision editing efficiencies at 10 tomato loci

We decided to evaluate the ability of the PE system to edit genomic sites in tomato by *Agrobacterium*-mediated stable transformations. The tomato transformation system was efficiently used for GT experiments in our laboratory (Fig. S2) [17]. We first attempted to edit 3 tomato loci—SlHKT1;2, SlEPSPS1, and SlOr—using PE2 and PE3 approaches with the T-DNA and replicon systems (Figs. S3 and S4 and Data S1). Transformed explants collected at 10 dpt were subjected to targeted deep sequencing of the targeted sites. However, in 2 replicates, we could not reveal any evidence of PE activity that was higher than the background level of the targeted sequencing method (Table 1). PE activity was shown to be locus specific, and several parameters, such as PBS and RT template lengths, may also affect PE efficiency in plants [6]. During our study, several PE data were reported that showed comparable PE efficiencies among the PE2, PE3, and PE3b constructs [6–8,10], and in some cases, the PE2 approach performed better than the PE3 approach [12]. We then extended the PE experiment to 7 more loci using the PE2 approach and single replicon-based vectors (Fig. 1A and Table S3) to check whether the PE system would work in tomato. Most of the loci were selected to require only single-nucleotide changes, and the RT template lengths were designed within the optimal ranges shown in reports on monocots. The PBS lengths were also appropriately selected (Table 1). Using targeted deep sequencing analysis, we found a higher PE performance at WUSCHEL HOMEOBOX 9 (SlWH9) (0.110%), KNOTTED1-LIKE HOMEOBOX PROTEIN1 (SlKD1) (0.018%), alcobaca (SlALC) (0.042%), and acetolactate synthase 1 (SlALS1) (0.095%) than at the mock control (Table 1). The low PE efficiency might be partially explained by the sample type (cotyledon explants) we collected for targeted deep sequencing. Since the cotyledon explants (0.1 × 0.3 cm in size) contained many untransformed cells because of the nature of the *Agrobacterium*-mediated transformation, they confer much lower editing efficiency than the protoplast system. Therefore, we also analyzed plants regenerated from the transformed cotyledon explants. We detected only low levels of PE alleles in several plants transformed with the SlALC and SlALS1 PE constructs (Fig. 1C and D). Sanger sequencing data revealed that only 1% of the PE alleles were successfully fixed in the plant genomes. Nevertheless, the PE efficiency obtained at the plant stage in our work is comparable to that of recently published data on tomato [15]. Moreover, in 1 event at SlALC, 2 indel byproducts were also formed, with frequencies of 1% and 3% (Fig. 1D).

**Table.** Prime editing (PE) efficiency in tomato using tobacco codon-optimized RT and rice codon-optimized PPE. nt, nucleotide; bp, base pairs.

No.	Locus	Targeted change	PBS length (nt)	RT length (nt)	Second nick position	Mock control		nCas9–RT		nCas9-PPE	
						Total reads	PE-mediated precise editing efficiency (%)	Total reads	PE-mediated precise editing efficiency (%)	Total reads	PE-mediated precise editing efficiency (%)
1	SlHKT1;2	N217D: +21 T to C; +10 A to G (reverse complement)	12	26	+76 (PE3)	321,183	0.007	104,610	0.000	-	-
			12	26	PE2			151,412	0.000	312,118	0.000
2	SlEPSPS1	T178I: +16 TG to GA; P182S: +4 CGG to AGA (reverse complement)	15	20	−55 (PE3)	2,526	0.000	1,357	0.000	-	-
			15	20	PE2			1,380	0.000	351,540	0.000
3	SlOr	R95H: +1 AGG to CAT	12	14	+61 (PE3)	449,607	0.000	140,325	0.000	-	-
			12	14	PE2			155,826	0.000	322,070	0.000
4	SlMBP21	Premature Q108stop: +1 C to T	13	13	PE2	49,811	0.018	57,943	0.022	148,426	0.019
5	WH9	G69D (s-classic): +6 G to A	13	14	PE2	59,484	0.018	54,735	0.110	202,599	0.032
6	SlKD1	1 bp deletion at 1,266 bp upstream of an open reading frame (ORF): +21 A del	13	26	PE2	44,911	0.002	60,765	0.018	169,327	0.008
7	SlPRD	A46 deletion: DELLAVLG to DELLVLG: +18 CTG del	13	22	PE2	46,727	0.000	50,399	0.000	130,662	0.000
8	SlALC	ALC to alc allele: T to A; V106D: +2 A to T (reverse complement)	13	12	PE2	51,754	0.015	45,543	0.042	159,889	0.064
9	SlDMR6-1	Premature truncation of the protein; E140stop: +1 G to T	13	13	PE2	37,532	0.128	50,231	0.020	170,289	0.021
10	SlALS1	P186S: +1 C to T	13	13	PE2	56,496	0.057	63,128	0.095	201,599	0.095

The low PE efficiency obtained for 10 tomato loci based on various editing types under our experimental conditions indicates that many critical factors affected the overall performance of PE in tomato and other dicot plants, as also concluded previously [13,14]. Wang and coworkers [13] reported PE efficiency at 0.06% ± 0.03% for correcting a mutated allele of the avrRpt2 gene of the bacterial plant pathogen *Pseudomonas syringae* that was co-infiltrated with agrobacteria carrying PE tool in tobacco leaves, and 0.07% ± 0.12% at a genomic site in *Arabidopsis* protoplasts. Similarly, Perroud and coworkers [14] obtained the highest PE efficiency at 0.06% of the transformed *Physcomitrium patens* protoplasts, and they showed that PE2 was functional in potato but at low frequency, and most edited plants were mosaic. To exclude the possibility that our tobacco codon-optimized RT coding sequence may trigger aberrant transcription or cryptic splicing, thereby affecting nCas9–RT activity, next, we used the nCas9-PPE coding sequence that showed good PE activities in rice and wheat [6]. The nCas9-PPE prime editor is a rice codon-optimized RT that performed the best among the RTs tested by Lin and coworkers [6]. We constructed PE2 tools using nCas9-PPE with all 10 tomato loci tested earlier (Figure 1b). However, targeted deep sequencing data collected for 2 replicates showed similar levels of PE activity across all 10 loci. The PE efficiency was even reduced in the case of the SlWH9 and SlKD1 loci but improved at the SlALC locus (Table 1), indicating that our tobacco codon-optimized RT activity was not worse than the reported version. These data illustrate that the PE approach needs improvement for further applications in tomato.

### The PE components negatively affect double-strand DNA cleavage

In an attempt to find solutions for improving the PE performance, we sought to investigate the roles of the PE components (i.e., the nCas9–RT and the pegRNAs) in the activation of the PE nuclease complex and nicking the template strand. We constructed binary plasmids with fully functional SpCas9 fused with RT or SpCas9 alone in combination with either pegRNAs or normal sgRNAs for 4 loci (MADS-box protein (SlMBP21), SlALC, SlDMR6-1, and SlALS1) (Fig. S5) and assessed their ability to generate indel mutations by targeted deep sequencing. Unexpectedly, the Cas9–RT fusion combined with the pegRNAs did not show indel mutation efficiencies above background levels at 3 (SlBPM21, SlDMR6-1, and SlALS1) out of 4 four loci (Fig. 1E), while the conventional Cas9-sgRNA complexes worked well at all the tested loci and significantly higher at the SlDMR6-1 and SlALS1 loci (Fig. 1E and Table S4). Cas9–RT combined with normal sgRNAs generated indel mutations at 2 of the 4 tested loci (SlMBP21 and SlALC), with indel mutation efficiencies similar to those of Cas9 and normal gRNAs (Fig. 1E and Table S4). The pegRNAs also supported the formation of indel mutations when combined with the Cas9 nuclease at 3 out of the 4 tested loci (SlMBP21, SlALC, and SlDMR6-1), but the indel mutation efficiencies were much lower compared to that of the Cas9/sgRNA complexes (Fig. 1E and Table S4). Either of the PE components alone worked at extremely lower efficiencies compared to Cas9 with normal gRNA at the ALS1 locus. These data indicate that the PE components negatively affected the cleaving activity of the Cas9 nuclease and that the use of pegRNAs led to reduced activity of the Cas9 enzyme (Fig. 1E). We reason that the Cas9–RT fusion might reduce the accessibility of the larger active complexes (Cas9–RT is approximately 236 kDa, compared to ~158 kDa for SpCas9 alone) to some genomic contexts, such as SlDMR6-1 and SlALS1, thereby blocking their cleavage activity. Moreover, the pegRNAs shared intramolecular complementarity between PBS and spacer sequences that potentially altered the secondary structure of the sgRNAs (Fig. S6), leading to possible interference of SpCas9 activation and/or the ability of gRNA to bind to targeted sequences and cleave them. The failure of the Cas9-RT/pegRNA complexes to produce indel mutations at 3 out of 4 loci and very low efficiency at the other locus might have resulted from the combined negative impacts of both components. On the basis of these data, we hypothesize that the activity of nCas9-RT/pegRNA complexes was affected by the negative impacts of both RT fusion and pegRNA structure, which led to extremely low PE efficiency, and by some unknown factor, resulting in especially low PE efficiencies in tomato and other dicot plants [13–15]. Unfortunately, similar evidence showing the negative impacts of the Cas9–RT fusion and the self-complementary structure of pegRNAs on the cleaving activity of the Cas9 nuclease has not been evaluated in other dicot plants. Nevertheless, we assume that the negative impacts of the PE components on its activities may be due to a conservative factor in dicot plants, leading to the low efficiency observed in this study and others [13–15].

### Modifications of the PBS did not significantly improve PE efficiency at 3 tested loci

If the above assumption is true, then we expect that, when the PBS is shortened, the PE efficiency will be improved. However, the PE data in plants suggested optimal ranges of PBS lengths that could not be too short to prime RT polymerization [6]. Higher incubation temperatures may help to destabilize the PBS–spacer intramolecular interactions, but they may also affect the activity of nuclease complexes or the activity of its target binding, as observed with the transient BE data (Fig. S1). That is also why Lu and coworkers [15] could not obtain a significant improvement in PE activity when they incubated explants at high temperatures. During the preparation of this manuscript, Lin and coworkers [19] reported that the optimal melting temperature (Tm) of the PBS was around 30 °C for PE in rice, reasoning that the PBS Tm affects the priming activity. Thus, we hypothesize that a range of optimized Tm exists for an active PBS of pegRNA in tomato. Alternatively, introducing several mismatches between the PBS and the spacer sequences may significantly weaken the interaction and improve PE activity in plants.

**Fig. 2.**
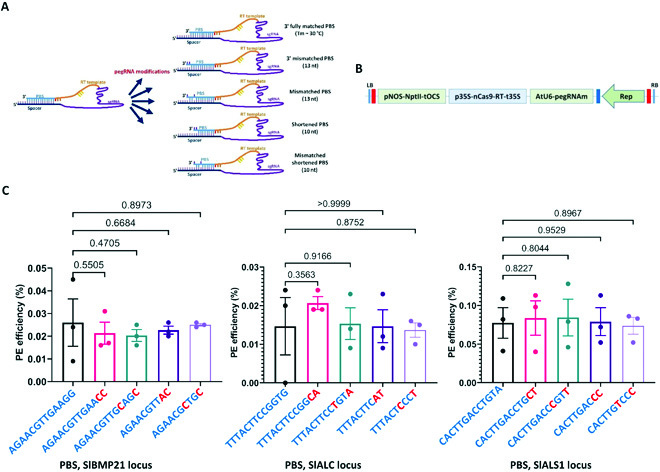
Assessment of PE efficiency using modified PBSs. (A) Modifications of the previously tested pegRNAs by shortening and/or introducing mismatches to their PBSs. Detailed modification of the pegRNAs at the tested loci is described in Fig. S7. (B) PE2 PE constructs using the modified pegRNAs. (C) Scatter dot–bar plots showing the pairwise comparisons between the PE efficiencies with the original and modified pegRNAs at the SlMBP21, SlALC, and SlALS1. The comparison at each locus is presented with the locus name at the bottom of the plot. The PBSs are shown on the bottom of each bar plot, with the red font letters representing mismatches between the PBS and spacer sequences. The sequences flanking the targeted sites were amplified at 10 dpt and analyzed by targeted deep sequencing in 3 replicates. The PE efficiencies were revealed by the RGEN Cas-Analyzer. Multiple comparisons of the means and plotting were conducted by GraphPad Prism version 9 using uncorrected Fisher’s LSD test. The *P* values of each compared mean pair are shown on the top of the bars

To test the hypothesis, we modified the pegRNAs of the three loci (SlMBP21, SlALC, and SlALS1) by introducing mismatches to the original or shortened PBSs (Fig. 2A). Total of 4 different modifications to an original PBS were made, and binary plasmids with replicon were similarly constructed (Fig. 2A and B) to the original PE vectors (Fig. 1A) using the tobacco codon-optimized RT. PE efficiency was assessed by targeted deep sequencing. In 3 replicates, the PE efficiencies at all the 3 tested loci were not significantly improved compared to that of the original pegRNAs (Fig. 2C and Table S5). These data were revealed even with the modified PBSs with Tm of 30, 32, or 34 °C compared to the original Tm of 38 °C (Fig. 2C and Table S5). To determine whether the mismatch would decrease the binding ability of PBSs and, thus, compromise the activity, we included another pegRNA with exactly matched PBS that has Tm at around 30 °C for each of the loci in an additional comparison experiment (Fig. 2A). However, targeted deep sequencing data revealed similarly low PE efficiencies compared to the pegRNAs with mismatches (Table S6). We reason that the mismatches introduced to either the PBSs or the shortened PBSs might not be sufficient to destabilize the self-complementarity predicted (Fig. S6). Another possibility was that the introduced mismatches were not sufficient to overcome the compromised activity of the CRISPR/Cas complexes since their activities were also seriously affected by the nCas9–RT fusion (Fig. 1). It is still unknown whether the way of introducing the mismatches was not perfectly matched with the criteria of the PBS Tm optimization in the case of the data shown by Lin and coworkers [19]. These data indicate that there are still more works need to be done

Since both the pegRNA and Cas9–RT fusions affected the cleaving activity of the complex, we can also use a 2-component PE complex like the MS2-MS2 coat protein (MCP) system [20] that separates the nCas9 and RT polypeptides to reduce the space constraint caused by the nCas9–RT fusion. However, the 2-component system may pose another risk for the proper assembly of the 2 components in a targeted genomic context, and in fact, split PE construction did not significantly improve PE efficiency in human cells [21]. Combination of multiple strategy for enhancing the expression and stability of the PE components would be the key to improve PE in dicotyledon plants as shown in monocots [22]. The PE performance was shown to be negatively affected by the mismatch repair pathway in human cells but could not be efficiently employed to improve PE in rice [23]. We also tested the MS2-MCP split system for separating nCas9 and RT enzymes and using SlMSH2DN (Solyc06g069230 (G671D), a dominant-negative version of a tomato MutS homolog 2 protein [24] that is an important component of mismatch repair) but obtained no improvement of PE activity in tomato (data not shown). Moreover, note that our delivery system for PE tools used geminiviral replicons that supposed to dramatically enhance the expression of PE components in tomato cells as shown earlier in our GT works [17] and others’ works [25]. Significant PE efficiency improvement in mammalian cells using PEmax and engineered pegRNAs [26] protected by trimmed evopreQ1 [27] could be simultaneously applied to solve the issues in dicot plants, as recently shown in rice [22]

Taken together, the PE approach offers an excellent alternative to precision gene editing in plants. However, we could not obtain a better editing efficiency than that of the BE tools in a transient assay in tobacco. Similar results were also recorded for 10 tomato loci using stable *Agrobacterium*-mediated transformations. We revealed low but comparable PE efficiency at the plant stage compared with recent data in plants. Further analyses showed that the PE components (nCas9–RT fusion and pegRNA) might largely contribute to its low activity in tomato. However, our PBS modifications for reducing Tm failed to improve the PE efficiency. Significant improvement to the PE approach needs to be made for further applications in tomato, and our data shed light on potential solutions for the improvement of this editing tool.

## Data Availability

Raw data used to support the findings presented in this study are available from the corresponding authors upon request.

## Supplementary Materials

Supplementary 1 Fig. S1. Failure of prime editing in tobacco cells in transient assays. Fig. S2. *Agrobacterium*-mediated transformation protocol used in this work. Fig. S3. PE targeting maps of the first 3 loci. (A) SlHKT1;2. (B) SlEPSPS1. (C) SlOr. Fig. S4. PE constructs for editing the SlHKT1;2, SlEPSPS1, and SlOr loci by PE2 and PE3 approaches. Fig. S5. Constructs used for the assessment of indel mutation formation by different combinations of either Cas9–RT or Cas9 with either sgRNAs or pegRNAs at the SlMBP21, SlALC, SlDMR6, and SlALS1 loci. Fig. S6. MFOLD-predicted secondary structures of pegRNAs and sgRNAs of SlMBP21, SlALC, SlDMR6-1, and SlALS1. Table S1. Sequences and primers used in this study. Table S2. Base editing efficiency obtained in the tobacco leaf infiltration assay. Table S3. PE targets in tomato used in the study. Table S4. Comparisons of indel mutation efficiency of the Cas9 complexes containing the PE components compared to the normal complex. Table S5. Comparisons of indel mutation efficiency of the Cas9 complexes containing the PE components compared to the normal complex. References [28–35].
