# Supplementary material for: The Obstacles and Potential Solution Clues of Prime Editing Applications in Tomato
Source: Biodes Res. 2022 Dec 15;2022:0001. doi: 10.34133/bdr.0001 (PMC10593121; doi:10.34133/bdr.0001)
Supplement: Supplementary 1 — Fig. S1. Failure of prime editing in tobacco cells in transient assays. Fig. S2. Agrobacterium-mediated transformation protocol used in this work. Fig. S3. PE targeting maps of the first 3 loci. (A) SlHKT1;2. (B) SlEPSPS1. (C) SlOr. Fig. S4. PE constructs for editing the SlHKT1;2, SlEPSPS1, and SlOr loci by PE2 and PE3 approaches. Fig. S5. Constructs used for the assessment of indel mutation formation by different combinations of either Cas9–RT or Cas9 with either sgRNAs or pegRNAs at the SlMBP21, SlALC, SlDMR6, and SlALS1 loci. Fig. S6. MFOLD-predicted secondary structures of pegRNAs and sgRNAs of SlMBP21, SlALC, SlDMR6-1, and SlALS1. Table S1. Sequences and primers used in this study. Table S2. Base editing efficiency obtained in the tobacco leaf infiltration assay. Table S3. PE targets in tomato used in the study. Table S4. Comparisons of indel mutation efficiency of the Cas9 complexes containing the PE components compared to the normal complex. Table S5. Comparisons of indel mutation efficiency of the Cas9 complexes containing the PE components compared to the normal complex. References [28–35]. [file 0001.f1.pdf]

The obstacles and potential solution clues of prime editing applications in tomato

Tien Van Vu[1,2,*], Ngan Thi Nguyen[1], Jihae Kim[1], Swati Das[1], Jinsu Lee[1], Jae-Yean Kim[1,3,4*]

[1]Division of Applied Life Science (BK21 FOUR Program), Plant Molecular Biology and Biotechnology Research Center, Gyeongsang National University, Jinju 660-701, Republic of Korea.

[2]National Key Laboratory for Plant Cell Biotechnology, Agricultural Genetics Institute, Km 02, Pham Van Dong Road, Co Nhue 1, Bac Tu Liem, Hanoi 11917, Vietnam.

[3]Division of Life Science, Gyeongsang National University, 501 Jinju-daero, Jinju 52828, Republic of Korea.

[4] Nulla Bio Inc, 501 Jinjudaero, Jinju 660-701, Republic of Korea.

***Correspondence: Tien Van Vu (tienvu.agi@gmail.com; ORCID: 0000-0002-6369-7664). Jae-Yean Kim (kimjy@gnu.ac.kr; ORCID ID: 0000-0002-1180-6232)**.

Keywords: Prime editor; CRISPR/SpCas9; Precision gene editing; Prime editing; DSB-independent gene editing, Geminiviral replicon.

SUPPLEMENTAL FIGURES, TABLES AND REFERENCES

Supplemental Figures, Tables and References

**Supplemental item**	**Page**
Supplemental Figure 1: Failure of prime editing in tobacco cells in transient assays	3-4
Supplemental Figure 2: *Agrobacterium*-mediated transformation protocol used in this work.	5
Supplemental Figure 3: PE targeting maps of the first three loci. (A) SlHKT1;2. (B) SlEPSPS1. (C) SlOr.	6
Supplemental Figure 4: PE constructs for editing the SlHKT1;2, SlEPSPS1 and SlOr loci by PE2 and PE3 approaches.	7
Supplemental Figure 5: Constructs used for the assessment of indel mutation formation by different combinations of either Cas9-RT or Cas9 with either sgRNAs or pegRNAs at the SlBMP21, SlALC, SlDMR6 and SlALS1 loci.	8
Supplemental Figure 6: MFOLD-predicted secondary structures of pegRNAs and sgRNAs of SlBMP21, SlALC, SlDMR6-1 and SlALS1.	9-10
Supplemental Figure 7. Modifications of the pegRNAs for improving PE performance	11
Supplemental Table 1. Sequences and primers used in this study.	12-13
Supplemental Table 2. Base editing efficiency obtained in the tobacco leaf infiltration assay.	14
Supplemental Table 3. PE targets in tomato used in the study.	15
Supplemental Table 4. Comparisons of indel mutation efficiency of the Cas9 complexes containing the PE components compared to the normal complex.	16
Supplemental Table 5. Comparisons of indel mutation efficiency of the Cas9 complexes containing the PE components compared to the normal complex.	17
Supplemental Table 6. Targeted deep sequencing data revealed from PE tools using shortened PBS and mismatched PBS	18
Supplemental references	19

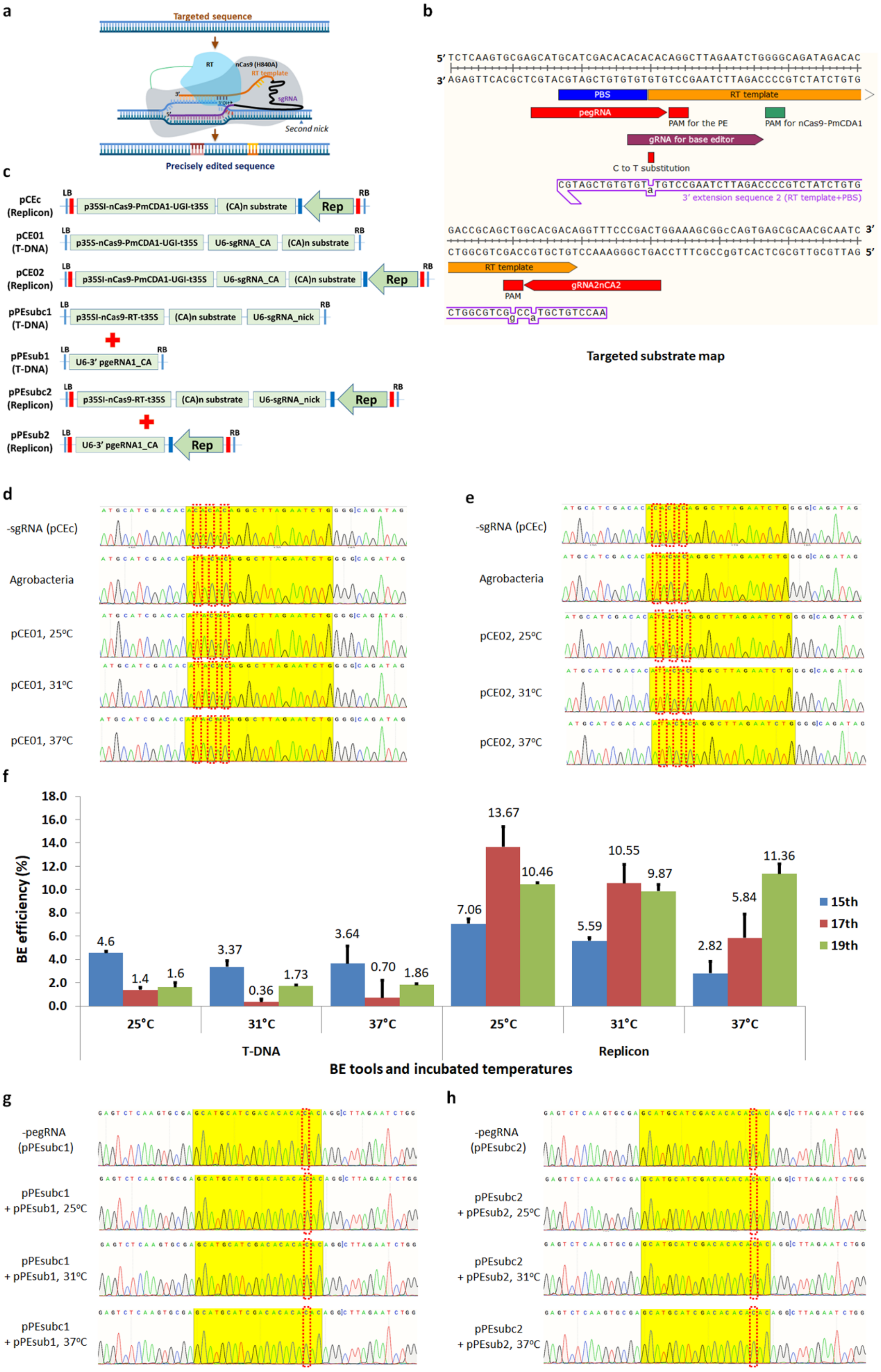

a
Targeted sequence
RT
nCas9 (H840A)
RT template
sgRNA
Second nick
Precisely edited sequence
b
5' TCTCAAGTGCGAGCATGCATCGACACACACACAGGCTTAGAATCTGGGGCAGATAGACAC
3' AGAGTTCACGCTCGTACGTAGCTGTGTGTGTGTCCGAATCTTAGACCCCGTCTATCTGTG
PBS
RT template
pegRNA
PAM for the PE
PAM for nCas9-PmCDA1
gRNA for base editor
C to T substitution
3' extension sequence 2 (RT template+PBS)
GACCGCAGCTGGCACGACAGGTTTCCCGACTGGAAAGCGGCCAGTGAGCGCAACGCAATC 3'
CTGGCGTCGACCGTGCTGTCCAAAGGGCTGACCTTTCGCCGGTCACTCGCGTTGCGTTAG 5'
RT template
gRNA2nCA2
PAM
Targeted substrate map
c
pCEc (Replicon)
p35SI-nCas9-PmCDA1-UGI-t35S
(CA)n substrate
Rep
pCE01 (T-DNA)
p35S-nCas9-PmCDA1-UGI-t35S
U6-sgRNA_CA
(CA)n substrate
pCE02 (Replicon)
p35SI-nCas9-PmCDA1-UGI-t35S
U6-sgRNA_CA
(CA)n substrate
Rep
pPEsubc1 (T-DNA)
p35SI-nCas9-RT-t35S
(CA)n substrate
U6-sgRNA_nick
pPEsub1 (T-DNA)
U6-3' pgeRNA1_CA
pPEsubc2 (Replicon)
p35SI-nCas9-RT-t35S
(CA)n substrate
U6-sgRNA_nick
Rep
pPEsub2 (Replicon)
U6-3' pgeRNA1_CA
Rep
LB
RB
d
-sgRNA (pCEc)
Agrobacteria
pCE01, 25ºC
pCE01, 31ºC
pCE01, 37ºC
e
-sgRNA (pCEc)
Agrobacteria
pCE02, 25ºC
pCE02, 31ºC
pCE02, 37ºC
f
BE efficiency (%)
18.0
16.0
14.0
12.0
10.0
8.0
6.0
4.0
2.0
0.0
4.6
1.4
1.6
3.37
0.36
1.73
3.64
0.70
1.86
7.06
13.67
10.46
5.59
10.55
9.87
2.82
5.84
11.36
15th
17th
19th
25°C
31°C
37°C
25°C
31°C
37°C
T-DNA
Replicon
BE tools and incubated temperatures
g
-pegRNA (pPEsubc1)
pPEsubc1 + pPEsub1, 25ºC
pPEsubc1 + pPEsub1, 31ºC
pPEsubc1 + pPEsub1, 37ºC
h
-pegRNA (pPEsubc2)
pPEsubc2 + pPEsub2, 25ºC
pPEsubc2 + pPEsub2, 31ºC
pPEsubc2 + pPEsub2, 37ºC

Supplemental Figure 1: Failure of prime editing in tobacco cells in transient assays. (a) Schematic processes of primed editing. The nontargeted strand is nicked by CRISPR/Cas9 nickase (H840A), and the 3’ nicked end binds to a complementary RNA (priming binding site, PBS, 13-15 nt) introduced by extending the 3’ end of the sgRNA (RT template) and primes reverse transcription by the nCas9-fused RT using its free OH group. RT copies the genetic information from the RNA template that is complementary to the sequence downstream from the nicked site and includes intentionally introduced base modifications (indicated by red and bright orange lines). The RT product appears as a 3’-flap sequence that competes with the full complement of the original 5’-flap during the repair process, and a precisely edited sequence could be fixed to the targeted site after 5’-flap removal and subsequent replication of the DNA. A second nick is introduced during the improvement of the prime editor for supporting 3’-edited flap fixation. (b) Selected synthetic target and pegRNA design. pegRNA and its 3’ extension and second nick spacer sequences are indicated. A PmCDA1-based C->T editing tool is designed in parallel for comparison, and its spacer sequence is also shown. Intended base modifications are denoted and explained. (c) Vector arrangements for the study. The nCas9(D10A)-PmCDA1 base editing system is designed with the control plasmid (pCEc, -gRNA), T-DNA (pCE1), and replicon (pCE2)-based editors. The prime editors are designed with a dual vector system for T-DNA (pPEsubc1 + pPEsub1) and replicon (pPEsubc2 + pPEsub2)-based tools for editing C->T and C->A (shown in b). (d-e) Sequencing data showing C->T editing by T-DNA- (d) and replicon-based (e) tools (Supplemental Table 2, (e)). The –sgRNA (pCEc) chromatogram represents all the temperature tested. (f) Editing efficiency of the base editors. The T-DNA construct represents the pCE1 plasmid, and the replicon construct is for the pCE2 plasmid. The editing efficiencies at different temperatures are plotted for the 15th, 17th, and 19th bases counted from the PAM site to its upstream spacer sequence. (g-h) Failure of the T-DNA- (g) and replicon-based (g) prime editors to edit the intended bases. The -pegRNA (pPEsubc1) and -pegRNA (pPEsubc2) chromatograms represent for all the temperature tested. Discontinuous red boxes denote the intended modifications in each of the editing conditions (25, 31, and 37°C) for the editors and the control.

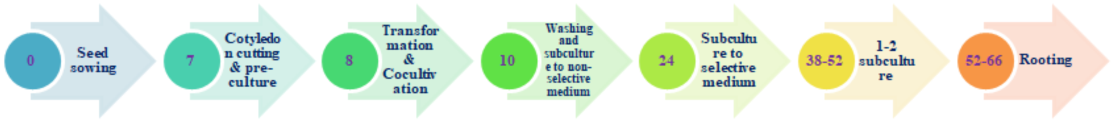

Stage	Germination	Pre-culture, callus induction	Co-cultivation, callus induction	Selection, callus induction and shoot regeneration	Selection, callus induction and shoot regeneration	Selection, shoot formation and elongation	Selection, root formation
Medium	1/2MSO	PREMC	ABM-MS+AS	SEL4-80	SEL4-80	SEL4-80	RIM
Temperature (°C)	25±2	25±1	25±1	28±1 for 5 days and then 25±2	25±2	25±2	25±2
Photoperiod	3 days-dark and then 4days 16L/8D	1 day dark	2 days-dark	5 days-8L/16D and then 9 days-16L/8D	14 days 16L/8D	14 days 16L/8D	10-14 days 16L/8D
Data collection (if needed)	-	-			Sampling at 10dpt		Sampling at plant stage

Supplemental Figure 2: *Agrobacterium*-mediated transformation protocol used in this work. The step-by-step protocol is presented with each number in the circles indicating the number of days after seed sowing (upper panel), and the treatments used in each step are shown in the lower panel.

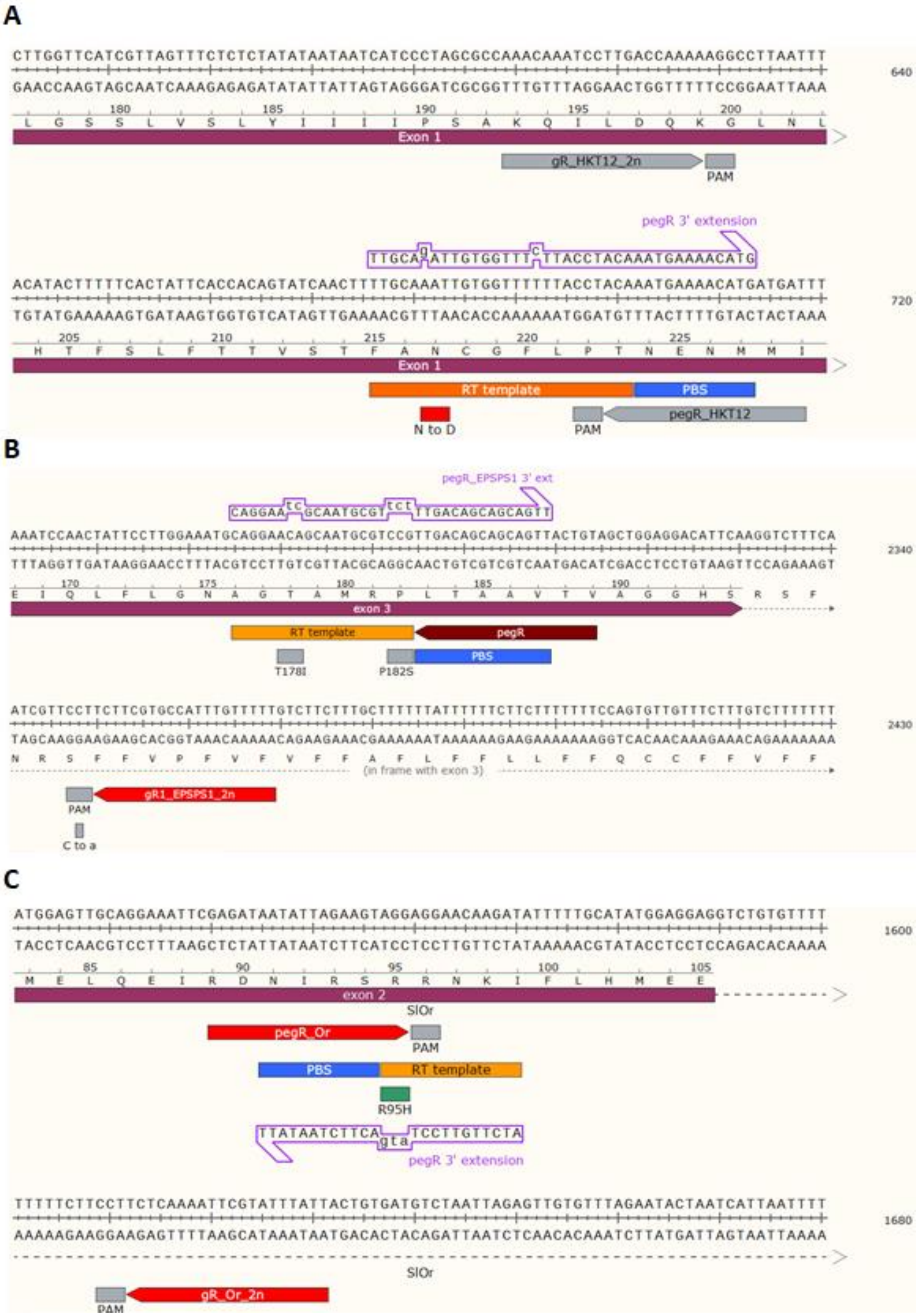

Supplemental Figure 3: PE targeting maps of the first three loci. (A) SlHKT1;2. (B) SlEPSPS1. (C) SlOr.

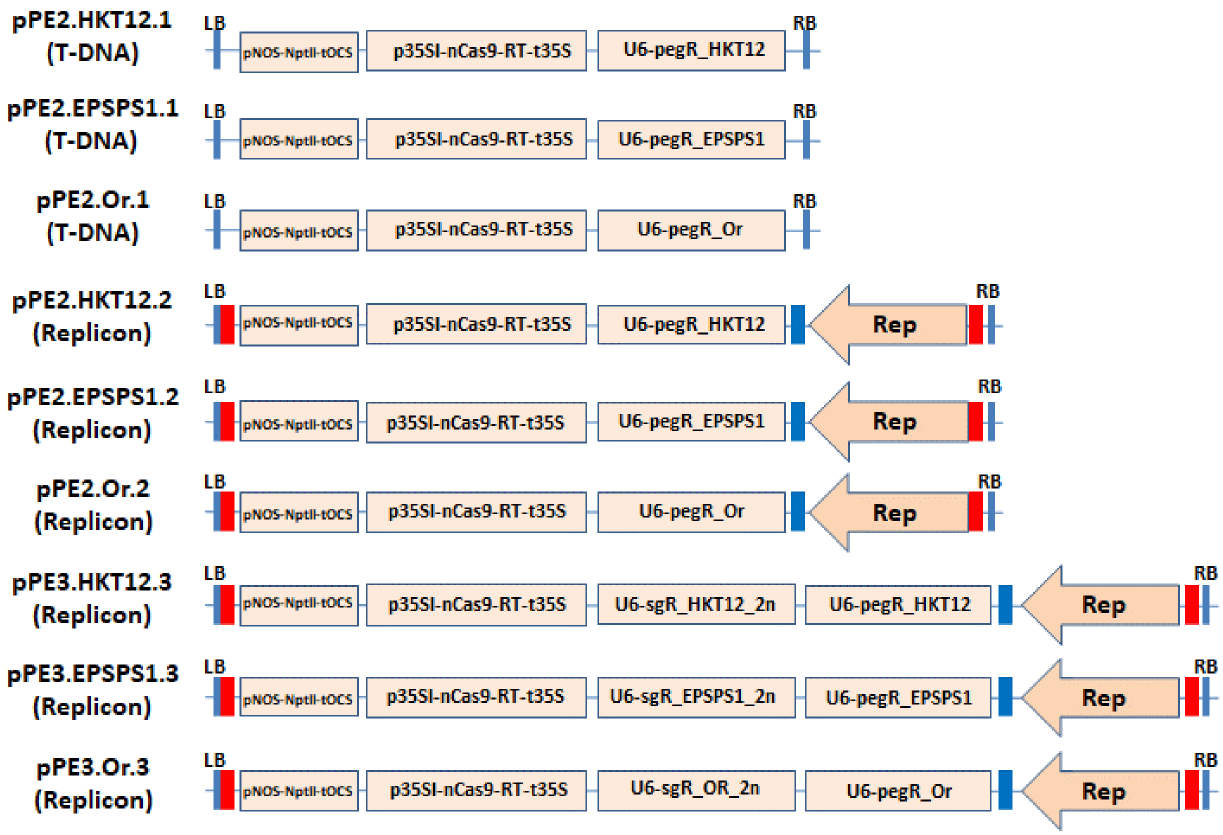

Supplemental Figure 4: PE constructs for editing the SlHKT1;2, SlEPSPS1 and SlOr loci by PE2 and PE3 approaches.

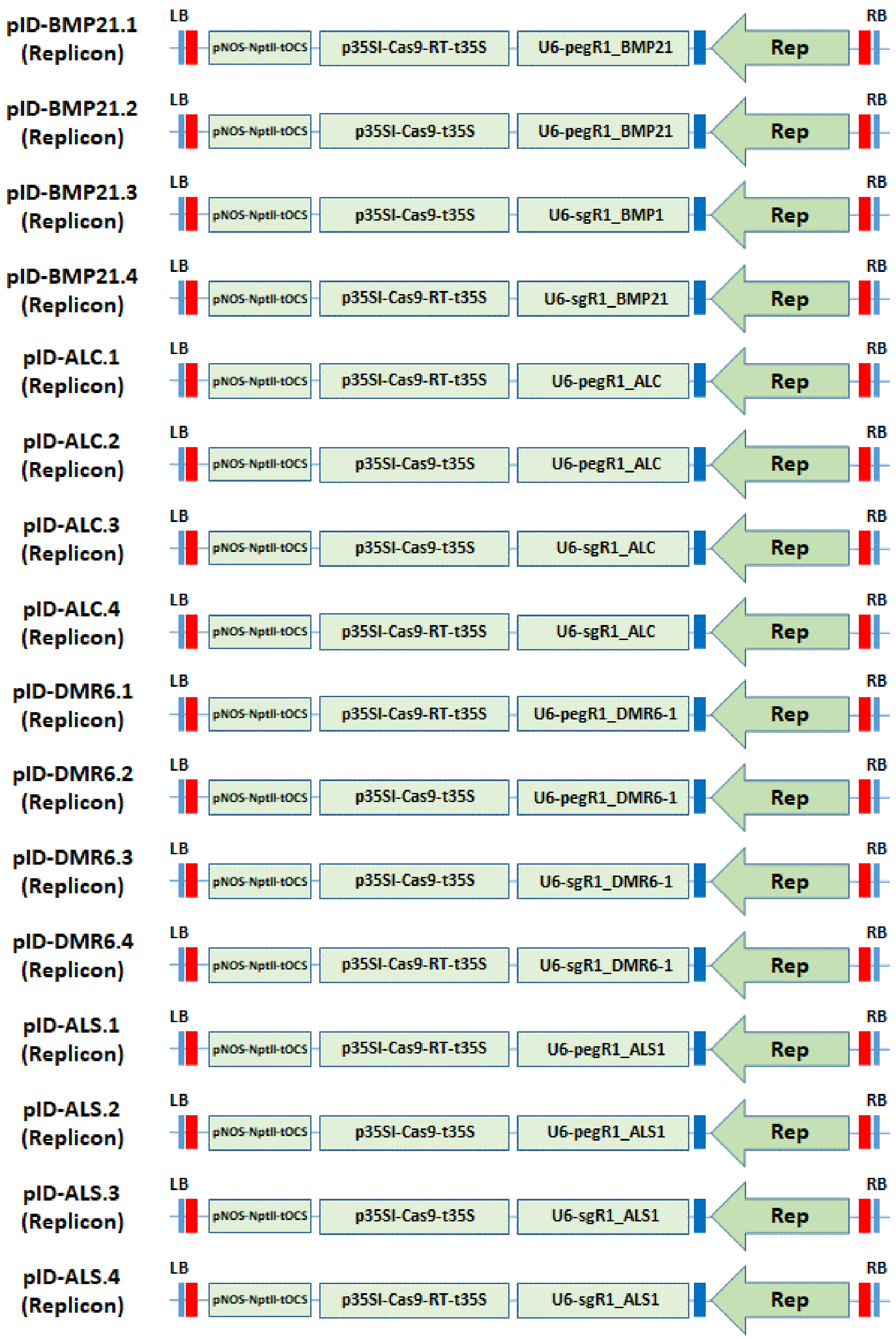

Supplemental Figure 5: Constructs used for the assessment of indel mutation formation by different combinations of either Cas9-RT or Cas9 with either sgRNAs or pegRNAs at the SlBMP21, SlALC, SlDMR6 and SlALS1 loci. All the component were expressed with the geminiviral replicon system.

	pegRNA secondary structure	sgRNA secondary structure
**SlBMP21**	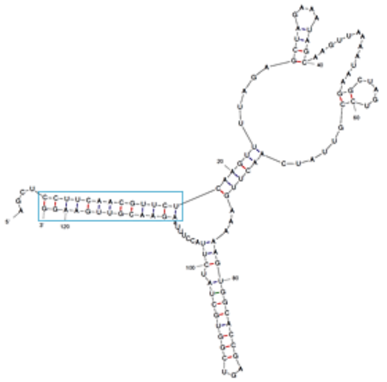<br>dG=-36.96 kcal/mol	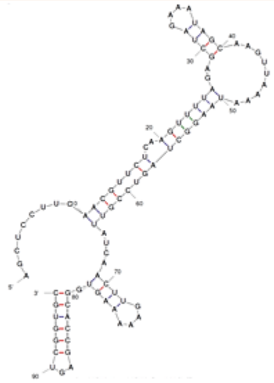<br>dG=-22.70 kcal/mol
**SlALC**	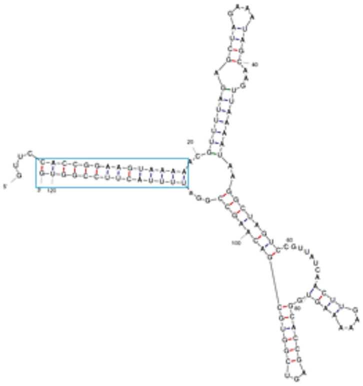<br>dG=-44.98 kcal/mol	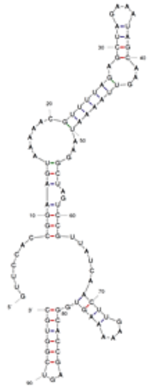<br>dG=-23.20 kcal/mol
**SlDMR6-1**	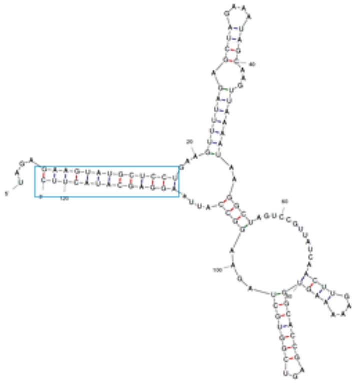<br>dG=-41.57 kcal/mol	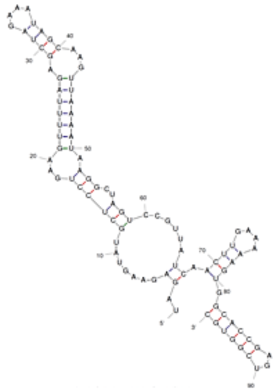<br>dG=-21.50 kcal/mol
**SlALS1**	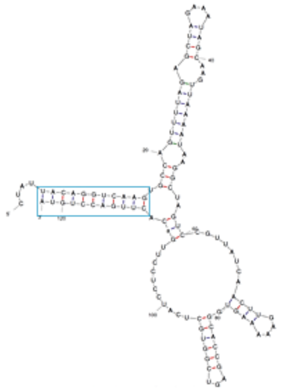<br>dG=-37.28 kcal/mol	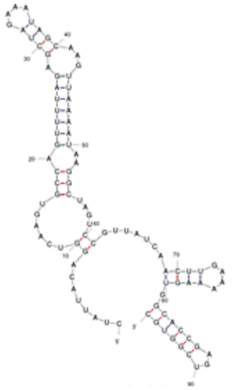<br>dG=-24.20 kcal/mol

Supplemental Figure 6: MFOLD-predicted secondary structures of pegRNAs and sgRNAs of SlBMP21, SlALC, SlDMR6-1 and SlALS1. The pegRNA structures (left panel) and sgRNA structures (right panel) were predicted using MFOLD software. The deltaG (dG) of each structure is denoted at the bottom of it. The discontinuous light blue boxes indicate the intermolecular annealing of the PBS and spacer sequences.

No.	Locus	pegR name	PBS (nt)	RT template	Tm of PBS-spacer (oC)	pegRNA (5'-3')
1		pegR.BMPm0	11	13	30	AGCTCCTTCAACGTTCTCAAGTTTTAGAGCTAGAAATAGCAAGTTAAAATAAGGCTAGTCC<br>GTTATCAACTTGAAAAAGTGGCACCGAGTCGGTGCTATCTTACCTTTaAGAACGTTGAA
2		pegR.BMPm1	13	13	30	AGCTCCTTCAACGTTCTCAAGTTTTAGAGCTAGAAATAGCAAGTTAAAATAAGGCTAGTCC<br>GTTATCAACTTGAAAAAGTGGCACCGAGTCGGTGCTATCTTACCTTTaAGAACGTTGAACC
3	SlBMP21	pegR.BMPm2	13	13	32	AGCTCCTTCAACGTTCTCAAGTTTTAGAGCTAGAAATAGCAAGTTAAAATAAGGCTAGTCC<br>GTTATCAACTTGAAAAAGTGGCACCGAGTCGGTGCTATCTTACCTTTaAGAACGTTGCAGC
4		pegR.BMPm3	10	13	22	AGCTCCTTCAACGTTCTCAAGTTTTAGAGCTAGAAATAGCAAGTTAAAATAAGGCTAGTCC<br>GTTATCAACTTGAAAAAGTGGCACCGAGTCGGTGCTATCTTACCTTTaAGAACGTTAC
5		pegR.BMPm4	10	13	24	AGCTCCTTCAACGTTCTCAAGTTTTAGAGCTAGAAATAGCAAGTTAAAATAAGGCTAGTCC<br>GTTATCAACTTGAAAAAGTGGCACCGAGTCGGTGCTATCTTACCTTTaAGAACGCTGC
6		pegR.ALCm0	11	12	32	GTTCCACCGGAAGTAAAAACGTTTTAGAGCTAGAAATAGCAAGTTAAAATAAGGCTAGTCC<br>GTTATCAACTTGAAAAAGTGGCACCGAGTCGGTGGACAAGCCGGaTTTTACTTCCGG
7		pegR.ALCm1	13	12	32	GTTCCACCGGAAGTAAAAACGTTTTAGAGCTAGAAATAGCAAGTTAAAATAAGGCTAGTCC<br>GTTATCAACTTGAAAAAGTGGCACCGAGTCGGTGGACAAGCCGGaTTTTACTTCCGGCA
8	SlALC	pegR.ALCm2	13	12	30	GTTCCACCGGAAGTAAAAACGTTTTAGAGCTAGAAATAGCAAGTTAAAATAAGGCTAGTCC<br>GTTATCAACTTGAAAAAGTGGCACCGAGTCGGTGGACAAGCCGGaTTTTACTTCCTGTA
9		pegR.ALCm3	10	12	20	GTTCCACCGGAAGTAAAAACGTTTTAGAGCTAGAAATAGCAAGTTAAAATAAGGCTAGTCC<br>GTTATCAACTTGAAAAAGTGGCACCGAGTCGGTGGACAAGCCGGaTTTTACTTCAT
10		pegR.ALCm4	10	12	22	GTTCCACCGGAAGTAAAAACGTTTTAGAGCTAGAAATAGCAAGTTAAAATAAGGCTAGTCC<br>GTTATCAACTTGAAAAAGTGGCACCGAGTCGGTGGACAAGCCGGaTTTTACTCCCT
11		pegR.ALSm0	10	13	30	CTATTACAGGTCAAGTGCCAGTTTTAGAGCTAGAAATAGCAAGTTAAAATAAGGCTAGTCC<br>GTTATCAACTTGAAAAAGTGGCACCGAGTCGGTGTCATCCTCCTTGaCACTTGACCT
12		pegR.ALSm1	13	13	34	CTATTACAGGTCAAGTGCCAGTTTTAGAGCTAGAAATAGCAAGTTAAAATAAGGCTAGTCC<br>GTTATCAACTTGAAAAAGTGGCACCGAGTCGGTGTCATCCTCCTTGaCACTTGACCTGCT
13	SlALS1	pegR.ALSm2	13	13	34	CTATTACAGGTCAAGTGCCAGTTTTAGAGCTAGAAATAGCAAGTTAAAATAAGGCTAGTCC<br>GTTATCAACTTGAAAAAGTGGCACCGAGTCGGTGTCATCCTCCTTGaCACTTGACCCGTT
14		pegR.ALSm3	10	13	24	CTATTACAGGTCAAGTGCCAGTTTTAGAGCTAGAAATAGCAAGTTAAAATAAGGCTAGTCC<br>GTTATCAACTTGAAAAAGTGGCACCGAGTCGGTGTCATCCTCCTTGaCACTTGACCC
15		pegR.ALSm4	10	13	26	CTATTACAGGTCAAGTGCCAGTTTTAGAGCTAGAAATAGCAAGTTAAAATAAGGCTAGTCC<br>GTTATCAACTTGAAAAAGTGGCACCGAGTCGGTGTCATCCTCCTTGaCACTTGTCCC

Supplemental Figure 7. Modifications of the pegRNAs for improving PE performance. At each locus, four modifications that introduce mismatches (purple font) to the original PBS sequences (13 nt in length, pegR.BMPm1, pegR.BMPm2, pegR.ALCm1, pegR.ALCm2, pegR.ALS1m1 and pegR.ALS1m2 for the BMP21, ALC, and ALS1) or shortened PBS (10nt, pegR.BMPm3, pegR.BMPm4, pegR.ALCm3, pegR.ALCm4, pegR.ALS1m3 and pegR.ALS1m4 for the BMP21, ALC, and ALS1) with mismatches. As controls, shortened and exactly matched PBS with Tm ~30°C (pegR.BMPm0, pegR.ALCm0, and pegR.ALSm0) were also used at each locus. The PBS and RT lengths and calculated Tm of the PBS-spacer pairing are also shown. In the pegRNA sequences, the red font letters denote spacer sequences; black font letters are SpCas9 gRNA scaffold sequences; orange font letters represent the RT template sequences; blue font letters are PBS sequences, and small red letters are introduced bases.

Supplemental Table 1. Sequences and primers used in this study

No.	Name	Sequence (5'-3')	Note
**Primers for PCR amplification of sequences flanking targeted sites**			
1	t35S-F1	TGTGTGAGTAGTTCCCAGATAAG	Sequences flanking the (CA)n substrate of pCEc and pCE02
2	RRA-R8	CTGACGAGGACTGGATGTTATC	
3	RB-qF2	CTCTTAGGTTTACCCGCCAATA	Sequences flanking the (CA)n substrate of pCE01
4	PmCDA1-R1	GATGGTTTCCGAGCACTATCA	
5	RT-F1	AGGCTAGAGGTAACCGGATG	Sequences flanking the (CA)n substrate of pEsubc1
6	PE-R1	TCGGTCACATGTGCATCCTC	
7	RT-F1	AGGCTAGAGGTAACCGGATG	Sequences flanking the (CA)n substrate of pEsubc2
8	PE-R2	CTGCGATATTACCGAGGACTAC	
9	t35S-sF1	TAGGGTTTCGCTCATGTGTTG	sequencing
**Primers for targeted deep sequencing sample preparation**			
9	HKPE-F1	ACAAAGATTATGAGCTAGGGAATGT	First PCR for SlHKT1;2
10	HKPE-R1	CCTTCAGAATTCCACTCCAATGA	
11	HKPE-F2	ACACTCTTTCCCTACACGACGCTCTTCCGATCT CGCCAAACAAATCCTTGACC	Second PCR for SlHKT1;2
12	HKPE-R2	GTGACTGGAGTTCAGACGTGTGCTCTTCCGAT CTTGAGGGATAAGAATGAGAAGAAGAC	
13	EPPE-F1	TGAGTTACTTACAACCTTGTGCT	First PCR for SlEPSPS1
14	EPPE-R1	GTAATTTATCACCAGGGCAACTATTATC	
15	EPPE-F2	ACACTCTTTCCCTACACGACGCTCTTCCGATCT GGCAGTTTCCTGTCGGTAA	Second PCR for SlEPSPS1
16	EPPE-R2	GTGACTGGAGTTCAGACGTGTGCTCTTCCGAT CTAAACAAATGGCACGAAGAAGG	
17	ORPE-F1	ATGCGAATCGTAGAAATGGAATTAG	First PCR for SlOr
18	ORPE-R1	ACTCGTTTCTTCGGTCCTTAAT	
19	ORPE-F2	ACACTCTTTCCCTACACGACGCTCTTCCGATCT AGGACCTGAGACAGTAGA	Second PCR for SlOr
20	ORPE-R2	GTGACTGGAGTTCAGACGTGTGCTCTTCCGAT CTAGACATCACAGTAATAAATACGA	
21	BMP-F1	CCGGTTAGTGATACTCAGGTATTG	First PCR for SlBMP21
22	BMP -R1	TGACTGCTTCCTAAGATGCTAAA	
23	BMP-F2	ACACTCTTTCCCTACACGACGCTCTTCCGATCT CTCGACGTTTTTCTCTTTCAACAGAT	Second PCR for SlBMP21
24	BMP-R2	GTGACTGGAGTTCAGACGTGTGCTCTTCCGAT CT CACTTATTAAAAACTTATGTAAATCTAAC	
25	WH9-F1	GGAGTCTATATAAGAAGTGTGTTAGGG	First PCR for SlWH9
26	WH9-R1	TCATTAAGAAGAAGTCCAGAAGAAGA	
27	WH9-F2	ACACTCTTTCCCTACACGACGCTCTTCCGATCT CAGAGCCAAAGCCTCGAT	Second PCR for SlWH9

28	WH9-R2	GTGACTGGAGTTCAGACGTGTGCTCTTCCGAT CTAGCATCACCTACTTGACCATATT	
29	KD1-F1	TTTGGTTACAACGTGTGAGATAAC	First PCR for SlKD1
30	KD1-R1	ACCATGTATAGTAGTCGTGTATGTT	
31	KD1-F2	ACACTCTTTCCCTACACGACGCTCTTCCGATCT ACACACAATCTGTAACAATGATAGTAAT	Second PCR for SlKD1
32	KD1-R2	GTGACTGGAGTTCAGACGTGTGCTCTTCCGAT CT CCTGACTAGTTTGAAGGGTATGT	
33	PRD-F1	GTGAATATTATTGCTGGCCTGTT	First PCR for SlPRD
34	PRD-R1	CAACTAGTACAACAGGTCTCGT	
35	PRD-F2	ACACTCTTTCCCTACACGACGCTCTTCCGATCT GGTGCTGTTTCTTCAGGGAA	Second PCR for SlPRD
36	PRD-R2	GTGACTGGAGTTCAGACGTGTGCTCTTCCGAT CT TAGCCATCTCAAGCTGTTCAAG	
37	ALC-F1	AAGTAGTGGACAAACATAAAGTAGTGG	First PCR for SlALC
38	ALC-R1	AATAATCTATGTCGAACCTCTTTCGG	
39	ALC-F2	ACACTCTTTCCCTACACGACGCTCTTCCGATCT ATATCCTAACGGGGCGAGGCCAAAT	Second PCR for SlALC
40	ALC-R2	GTGACTGGAGTTCAGACGTGTGCTCTTCCGAT CT GCATGATCCAATTAGTTTTTACCCCT	
41	DMR-F1	CTTTCAGGTTCTTATCGTTGGATTC	First PCR for SlDMR6-1
42	DMR-R1	AGGTTAGCTAGGTAGATTAGGTAGT	
43	DMR-F2	ACACTCTTTCCCTACACGACGCTCTTCCGATCT GGAGACAGTTCATAATTGGAGAGATTAT	Second PCR for SlDMR6-1
44	DMR-R2	GTGACTGGAGTTCAGACGTGTGCTCTTCCGAT CT TTGAAGTATGACTCAGATAGCA	
45	ALS-F1	CCTCACCATCTCCATGTTTCTC	First PCR for SlALS1
46	ALS-R1	GTCTCAGCTCCTCACTTGATTG	
47	ALS-F2	ACACTCTTTCCCTACACGACGCTCTTCCGATCT GCTACAAATCTTGTTAGTGGTCTTG	Second PCR for SlALS1
48	ALS-R2	GTGACTGGAGTTCAGACGTGTGCTCTTCCGAT CT GCTTCGTAATAGATCTCGTTACCTC	
49	HKT12-sF1	CAAAGATTATGAGCTAGGGAATGT	Sequencing primer for the SlHKT1;2 targeted site
50	EPSPS1-sF3	GGAGATATTTGTTAGAGTACCATCA	Sequencing primer for the SlEPSPS1 targeted site
51	WH9-sR1	CACCGAAGAAGTAGGAGAATTGA	Sequencing primer for the SlWH9 targeted site
52	ALC-sF1	CATGTGTTTAACGCAGCTAAGG	Sequencing primer for the SlALC targeted site
53	ALS1-sF1	CCCAGAAAGGGTTGTGATGT	Sequencing primer for the SlALS1targeted site

Supplemental Table 2. Base editing efficiency obtained in the tobacco leaf infiltration assay

Position counted from PAM	T-DNA			Replicon		
	**25°C**	**31°C**	**37°C**	**25°C**	**31°C**	**37°C**
15th	4.57 ± 0.14	3.37 ± 0.55	3.64 ± 1.5	7.06 ± 0.41	5.59 ± 0.25	2.82 ± 1.02
17th	1.38 ± 0.21	0.36 ± 0.22	0.70 ± 1.5	13.67 ± 1.74	10.55 ± 1.63	5.84 ± 2.03
19th	1.63 ± 0.41	1.73 ± 0.10	1.86 ± 0.03	10.46 ± 0.10	9.87 ± 0.57	11.36 ± 0.83

Supplemental Table 3. PE targets in tomato used in the study

No.	Gene/allele name	Accession no.	Genotype vs WT	Phenotype	Reference
1	*jointless-2/*SlMBP21	Solyc12g038510	L169Stop (T->A)	Absence of abscission layer in Pedicels/short pedicels	Roldan et al., 2017
2	WUSCHEL HOMEOBOX 9 (SlWH9)/*compound inflorescence (s)*	Solyc02g077390.1	G69D (s-classic)	Mutant alleles of s dramatically increase branch and flower number	Lippman et al., 2008
3	*Petroselinum (Pts) allele/KNOTTED 1-LIKE HOMEOBOX (KNOX)/SlKD1 (TOMATO KNOX-LIKE HOMEODOMAIN PROTEIN 1)*	*AF375969/* Solyc06g072480.1	1 bp deletion at 1266 bp upstream of the open reading frame (ORF)	A semidominant KD1 mutant in tomato with multiply compound leaves. TKD1 silencing delay absicion.	Kimura et al., 2008
4	PROCERA D/DELLA D allele (SlPRD)	Solyc11g011260	A46 deletion: DELLAVLG to DELLVLG	Dwarf/gibberellin-responsive dominant dwarf DELLA allele	Nir et al., 2017; Tomlinson et al., 2019
5	SlALC (Alcobaca)	FJ404469	ALC to alc allele: T to A; V106D	Long-shelf life	Garg et al., 2008
6	SlDMR6-1	Solyc03g080190.2	small deletions in the *SlDMR6-1* gene that result in frameshift and premature truncation of the protein	No significant detrimental effects in terms of growth and development under greenhouse conditions and show disease resistance against different pathogens, including *P. syringae*, *P. capsici* and *Xanthomonas* spp.	Thomazella et al., 2016
7	SlALS1 gene	Solyc03g044330	Any substitution to change Pro186 amino acid to Ser, Ala or Thr residues	Strong resistance to chlorsulfuron. After two weeks, explants were transferred onto a fresh culture medium with 40 ng/mL chlorsulfuron.	Yu et al., 2009

Supplemental Table 4. Comparisons of indel mutation efficiency of the Cas9 complexes containing the PE components compared to the normal complex.

Gene/allele name	Accession no.	Construction	PBS length (nt)	RT length (nt)	Total reads	Indel mutation efficiency (%)					p value** (Uncorrected Fisher's LSD)
						Replicate 1	Replicate 2	Replicate 3	Average	SEM	
SlBMP21	Solyc12g038510	-*	-	-	49819	0.026	-	-	0.026	-	-
		Cas9-RT + pegR_BMP21	13	13	132955	0.018	0.007	0.023	0.016	0.005	0.0877
		Cas9 + pegR_BMP21	13	13	139784	0.092	0.190	0.058	0.113	0.040	0.3248
		Cas9 + gR_BMP21	0	0	127696	0.355	0.238	0.089	0.227	0.077	
		Cas9-RT + gR_BMP21	0	0	128919	0.411	0.023	0.038	0.158	0.127	0.5375
SlALC	FJ404469	-	-	-	51907	0.075	-	-	0.075	-	-
		Cas9-RT + pegR_ALC	13	12	140946	0.119	0.108	0.093	0.107	0.007	0.2103
		Cas9 + pegR_ALC	13	12	140852	0.170	0.155	0.114	0.146	0.017	0.2748
		Cas9 + gR_ALC	0	0	129357	0.714	0.111	0.349	0.391	0.175	
		Cas9-RT + gR_ALC	0	0	121087	0.091	0.808	0.102	0.334	0.237	0.7896
SlDMR6-1	Solyc03g080190	-	-	-	37540	0.019	-	-	0.019	-	-
		Cas9-RT + pegR_DMR6-1	13	13	108382	0.010	0.021	0.020	0.017	0.004	0.0023
		Cas9 + pegR_DMR6-1	13	13	106969	0.254	0.161	0.025	0.147	0.067	0.0332
		Cas9 + gR_DMR6-1	0	0	124113	0.423	0.382	0.181	0.329	0.075	
		Cas9-RT + gR_DMR6-1	0	0	106991	0.006	0.017	0.019	0.014	0.004	0.0022
SlALS1	Solyc03g080190	-	-	-	56500	0.000	-	-	0.000	-	-
		Cas9-RT + pegR_ALS1	13	13	131194	0.000	0.032	0.023	0.018	0.009	<0.0001
		Cas9 + pegR_ALS1	13	13	145331	0.033	0.106	0.018	0.052	0.027	<0.0001
		Cas9 + gR_ALS1	0	0	144979	1.055	1.513	0.884	1.151	0.188	
		Cas9-RT + gR_ALS1	0	0	129222	0.039	0.057	0.031	0.042	0.008	<0.0001

*WT sample. **Multiple comparisons were conducted between the normal Cas9+gRNA combination and the complex containing either or both the PE components with the Uncorrected Fisher's LSD test using the Graphpad 9.0 software.

Supplemental Table 5. Comparisons of indel mutation efficiency of the Cas9 complexes containing the PE components compared to the normal complex.

Locus	PBS sequence (5'-3')	Tm of PBS (oC)	PE efficiency (%) Replicate 1	Replicate 2	Replicate 3	Average	SEM	p value* (Uncorrected Fisher's LSD)
SlBMP21	AGAACGTTGAAGG	38	0.024	0.009	0.045	0.026	0.010	
	AGAACGTTGAACC**	30	0.017	0.031	0.016	0.021	0.005	0.5505
	AGAACGTTGCAGC	32	0.020	0.016	0.025	0.020	0.003	0.4705
	AGAACGTTAC	22	0.022	0.020	0.026	0.023	0.002	0.6684
	AGAACGCTGC	24	0.026	0.024	0.025	0.025	0.001	0.8973
SlALC	TTTACTTCCGGTG	38	0.020	0.024	0.000	0.015	0.007	
	TTTACTTCCGGCA	32	0.019	0.024	0.019	0.020	0.002	0.3563
	TTTACTTCCTGTA	30	0.014	0.009	0.023	0.015	0.004	0.9166
	TTTACTTCAT	20	0.012	0.009	0.023	0.015	0.004	>0.9999
	TTTACTCCCT	22	0.010	0.015	0.016	0.014	0.002	0.8752
SlALS	CACTTGACCTGTA	38	0.109	0.082	0.041	0.077	0.020	
	CACTTGACCTGCT	34	0.098	0.113	0.040	0.084	0.022	0.8227
	CACTTGACCCGTT	34	0.128	0.079	0.046	0.084	0.024	0.8044
	CACTTGACCC	24	0.113	0.072	0.052	0.079	0.018	0.9529
	CACTTGTCCC	26	0.086	0.083	0.052	0.074	0.011	0.8967

*Multiple comparisons were conducted between PE complexes with the original PBS containing pegRNAs and that of the complexes containing modified PBS sequences with the Uncorrected Fisher's LSD test using the Graphpad 9.0 software. **red font: mismatches introduced between the PBSs and spacers.

Supplemental Table 6. Targeted deep sequencing data revealed from PE tools using shortened PBS and mismatched PBS

Locus	PBS sequence (5'-3')	Tm of PBS-spacer (oC)	Replicate 1			Replicate 2		
			Total reads	PE efficiency (%)	Indel efficiency (%)	Total reads	PE efficiency (%)	Indel efficiency (%)
BMP	AGAACGTTGAA	30	39192	0.015	0.013	22412	0.031	0.022
	AGAACGTTGAACC*	30	47378	0.019	0.015	25443	0.039	0.000
	AGAACGTTGCAGC	32	55099	0.015	0.018	27979	0.032	0.025
	AGAACGTTAC	22	52535	0.015	0.017	23439	0.026	0.013
	AGAACGCTGC	24	54017	0.020	0.015	26156	0.031	0.011
ALC	TTTACTTCCGG	32	50025	0.014	0.056	25835	0.012	0.081
	TTTACTTCCGGCA	32	52284	0.019	0.069	31389	0.019	0.076
	TTTACTTCCTGTA	30	38566	0.026	0.078	23446	0.013	0.060
	TTTACTTCAT	20	47192	0.030	0.087	26248	0.015	0.057
	TTTACTCCCT	22	46943	0.013	0.066	29077	0.014	0.079
ALS	CACTTGACCT	30	47374	0.103	0.017	29667	0.101	0.007
	CACTTGACCTGCT	34	47803	0.096	0.013	25106	0.068	0.028
	CACTTGACCCGTT	34	47225	0.104	0.017	26736	0.094	0.022
	CACTTGACCC	24	44622	0.083	0.011	26242	0.088	0.015
	CACTTGTCCC	26	41949	0.098	0.019	25926	0.093	0.004

*red font: mismatches introduced between the PBSs and spacers.
