# Supplementary material for: The Obstacles and Potential Solution Clues of Prime Editing Applications in Tomato
Source: Biodes Res. 2022 Dec 15;2022:0001. doi: 10.34133/bdr.0001 (PMC10593121; doi:10.34133/bdr.0001)
Supplement: Supplementary 2 — Data S1 file. [file 0001.f2.pdf]

SEQUENCES USED IN THE STUDY

- **Moloney Murine Leukemia Virus reverse transcriptase (MMLVrt) coding sequence (tobacco codon-optimized)**

  TCTGGAGGATCTAGCGGAGGATCCTCTGGGTCGGAAACACCAGGTACTAGCGAGTCAGCTACACCAGAGTCT
  AGTGGAGGCAGCAGCGGCGGGAGTAGCACACTAAATATAGAAGATGAATATCGGCTACATGAAACTTCAAA
  AGAGCCAGATGTTTCTCTAGGGAGCACATGGCTATCTGATTTTCCTCAAGCCTGGGCGGAAACCGGCGGCAT
  GGGACTGGCAGTTAGACAAGCTCCTCTGATTATACCACTGAAAGCAACATCTACTCCCGTTTCCATAAAACAAT
  ATCCCATGTCACAAGAAGCCAGATTAGGAATCAAGCCTCATATACAGAGACTGTTGGACCAGGGAATACTGG
  TACCCTGCCAGTCCCCTTGGAACACACCACTGCTACCCGTTAAGAAACCTGGTACTAATGATTATAGGCCTGTC
  CAAGATCTGAGAGAAGTCAATAAGCGTGTGGAAGATATTCACCCTACCGTGCCAAACCCTTACAACCTCTTGT
  CTGGACTACCACCGTCCCACCAATGGTACACTGTGCTTGATTTAAAGGATGCCTTTTTCTGTCTGAGACTCCAT
  CCCACAAGTCAGCCTCTTTTCGCCTTTGAATGGAGAGATCCAGAAATGGGGATTTCAGGACAATTGACCTGGA
  CTAGACTCCCACAGGGTTTCAAAAACAGTCCCACCCTGTTTAATGAGGCATTACACCGTGATTTGGCAGACTTT
  AGAATCCAGCACCCAGACTTGATTCTGCTACAGTACGTGGATGATTTACTGTTAGCCGCAACTTCTGAACTAG
  ACTGTCAACAGGGTACTCGAGCCCTGTTACAAACCTTGGGGAACCTTGGGTATCGGGCTTCAGCAAAGAAAG
  CTCAAATTTGCCAAAAACAAGTCAAGTATCTGGGGTATCTTCTAAAAGAGGGTCAGAGATGGCTTACTGAGG
  CAAGAAAAGAGACTGTGATGGGGCAGCCTACTCCGAAAACCCCTCGACAACTAAGGGAGTTCCTAGGGAAA
  GCAGGCTTCTGTCGCCTCTTCATCCCAGGATTTGCAGAAATGGCAGCCCCCCTGTACCCTCTCACCAAACCGG
  GTACTCTGTTTAATTGGGGCCCAGATCAACAAAAGGCTTATCAAGAAATTAAGCAAGCTTTGCTCACTGCCCC
  AGCCCTGGGTTTGCCAGATTTGACTAAGCCCTTTGAACTCTTTGTGGACGAGAAGCAAGGATACGCGAAAGG
  TGTTCTAACGCAAAAATTAGGACCTTGGCGTAGACCAGTGGCATACCTGTCAAAAAAATTGGATCCAGTTGCA
  GCTGGGTGGCCTCCTTGCCTAAGGATGGTAGCAGCCATTGCCGTACTGACAAAGGATGCCGGCAAGCTAACC
  ATGGGACAGCCACTAGTCATTCTGGCCCCACATGCAGTTGAAGCACTAGTCAAGCAACCTCCCGACCGCTGGC
  TTTCCAATGCGCGGATGACTCATTATCAGGCCTTACTTTTGGATACGGACCGGGTTCAGTTCGGACCGGTGGT
  AGCCTTAAACCCGGCTACGCTGTTACCACTGCCTGAGGAAGGACTACAACACAATTGTCTTGATATTTTAGCC
  GAAGCACACGGAACCCGACCCGATCTAACGGACCAGCCGCTCCCAGATGCCGATCATACCTGGTACACTGAT
  GGAAGCAGTTTGTTACAAGAGGGACAGCGTAAGGCGGGAGCTGCGGTTACCACCGAGACAGAGGTAATCTG
  GGCTAAAGCACTGCCAGCCGGTACATCCGCTCAGCGGGCTGAACTGATAGCACTCACCCAGGCACTAAAAAT
  GGCAGAAGGTAAGAAGCTAAACGTTTATACTGATAGCAGATATGCTTTTGCTACTGCTCATATCCATGGAGAA
  ATATACAGAAGGCGTGGTTGGCTCACATCAGAAGGCAAGGAAATCAAAAATAAAGACGAGATTTTGGCCCTA
  CTAAAAGCCCTCTTTCTTCCTAAAAGATTAAGCATAATTCATTGTCCAGGACATCAAAAAGGACACAGCGCCG
  AGGCTAGAGGTAACCGGATGGCTGATCAAGCTGCCCGAAAGGCAGCTATTACTGAGACTCCAGATACCTCTA
  CACTCCTCATTGAAAATTCATCACCTTCTGGCGGATCAAAAAGAACCGCCGACGGTTCGGAATTCGAACCAAA
  GAAGAAGAGGAAAGTGTAA

  Blue font: linker; purple font: MMLVrt; green font: SV40 NLS; black font: spacer sequence; red font: stop codon.

- **Moloney Murine Leukemia Virus reverse transcriptase (MMLVrt) coding sequence (rice codon-optimized) from Addgene Plasmid #140445.**
- **(CA)n substrate sequence**

TCTCAAGTGCGAGCATGCATCGACACACACACAGGCTTAGAATCTGGGGCAGATAGACACGACCGCAGCTGG
CACGACAGGTTTCCCGACTGGAAAGCGGcCAGTGAGCGCAACGCAATCCACCAGCGcC

- **p35SI-nCas9-PmCDA1-UGI-t35S**

GAATTCCAATCCCACAAAAATCTGAGCTTAACAGCACAGTTGCTCCTCTCAGAGCAGAATCGGGTATTCAACA
CCCTCATATCAACTACTACGTTGTGTATAACGGTCCACATGCCGGTATATACGATGACTGGGGTTGTACAAAG
GCGGCAACAAACGGCGTTCCCGGAGTTGCACACAAGAAATTTGCCACTATTACAGAGGCAAGAGCAGCAGCT
GACGCGTACACAACAAGTCAGCAAACAGACAGGTTGAACTTCATCCCCAAAGGAGAAGCTCAACTCAAGCCC
AAGAGCTTTGCTAAGGCCCTAACAAGCCCACCAAAGCAAAAAGCCCACTGGCTCACGCTAGGAACCAAAAGG
CCCAGCAGTGATCCAGCCCCAAAAGAGATCTCCTTTGCCCCGGAGATTACAATGGACGATTTCCTCTATCTTTA
CGATCTAGGAAGGAAGTTCGAAGGTGAAGGTGACGACACTATGTTCACCACTGATAATGAGAAGGTTAGCCT
CTTCAATTTCAGAAAGAATGCTGACCCACAGATGGTTAGAGAGGCCTACGCAGCAAGTCTCATCAAGACGATC
TACCCGAGTAACAATCTCCAGGAGATCAAATACCTTCCCAAGAAGGTTAAAGATGCAGTCAAAAGATTCAGG
ACTAATTGCATCAAGAACACAGAGAAAGACATATTTCTCAAGATCAGAAGTACTATTCCAGTATGGACGATTC
AAGGCTTGCTTCATAAACCAAGGCAAGTAATAGAGATTGGAGTCTCTAAAAAGGTAGTTCCTACTGAATCTAA
GGCCATGCATGGAGTCTAAGATTCAAATCGAGGATCTAACAGAACTCGCCGTCAAGACTGGCGAACAGTTCA
TACAGAGTCTTTTACGACTCAATGACAAGAAGAAAATCTTCGTCAACATGGTGGAGCACGACACTCTGGTCTA
CTCCAAAAATGTCAAAGATACAGTCTCAGAAGATCAAAGGGCTATTGAGACTTTTCAACAAAGGATAATTTCG
GGAAACCTCCTCGGATTCCATTGCCCAGCTATCTGTCACTTCATCGAAAGGACAGTAGAAAAGGAAGGTGGCT
CCTACAAATGCCATCATTGCGATAAAGGAAAGGCTATCATTCAAGATCTCTCTGCCGACAGTGGTCCCAAAGA
TGGACCCCCACCCACGAGGAGCATCGTGGAAAAAGAAGAGGTTCCAACCACGTCTACAAAGCAAGTGGATTG
ATGTGACATCTCCACTGACGTAAGGGATGACGCACAATCCCACTATCCTTCGCAAGACCCTTCCTCTATATAAG
GAAGTTCATTTCATTTGGAGAGGACACGCTCGAGTATAAGGTAAATTTCTGTGTTCCTTATTCTCTCAAAATCT
TCGATTTTGTTTTCGTTCGATCCCAATTTCGTATATGTTCTTTGGTTTAGATTCTGTTAATCTTAGATCGAAGAT
GATTTTCTGGGTTTGATCGTTAGATATCATCTTAATTCTCGATTAGGGTTTCATAGATATCATCCGATTTGTTCA
AATAATTTGAGTTTTGTCGAATAATTACTCTTCGATTTGTGATTTCTATCTAGATCTGGTGTTAGTTTCTAGTTT
GTGCGATCGAATTTGTCGATTAATCTGAGTTTTTCTGATTAACAGGAGCTCATTTTTACAACAATTACCAACAA
CAACAAACAACAAACAACATTACAATTACATTTACAATTATCGATACAATGCCCAAAAAGAAAAGAAAAGTGG
ACAAGAAGTACTCCATTGGGCTCGCTATCGGCACAAACAGCGTCGGCTGGGCCGTCATTACGGACGAGTACA
AGGTGCCGAGCAAAAAATTCAAAGTTCTGGGCAATACCGATCGCCACAGCATAAAGAAGAACCTCATTGGCG
CCCTCCTGTTCGACTCCGGGGAGACGGCCGAAGCCACGCGGCTCAAAAGAACAGCACGGCGCAGATATACCC
GCAGAAAGAATCGGATCTGCTACCTGCAGGAGATCTTTAGTAATGAGATGGCTAAGGTGGATGACTCTTTCTT
CCATAGGCTGGAGGAGTCCTTTTTGGTGGAGGAGGATAAAAAGCACGAGCGCCACCCAATCTTTGGCAATAT
CGTGGACGAGGTGGCGTACCATGAAAAGTACCCAACCATATATCATCTGAGGAAGAAGCTTGTAGACAGTAC
TGATAAGGCTGACTTGCGGTTGATCTATCTCGCGCTGGCGCATATGATCAAATTTCGGGGACACTTCCTCATC
GAGGGGGACCTGAACCCAGACAACAGCGATGTCGACAAACTCTTTATCCAACTGGTTCAGACTTACAATCAGC
TTTTCGAAGAGAACCCGATCAACGCATCCGGAGTTGACGCCAAAGCAATCCTGAGCGCTAGGCTGTCCAAATC
CCGGCGGCTCGAAAACCTCATCGCACAGCTCCCTGGGGAGAAGAAGAACGGCCTGTTTGGTAATCTTATCGC
CCTGTCACTCGGGCTGACCCCCAACTTTAAATCTAACTTCGACCTGGCCGAAGATGCCAAGCTTCAACTGAGC
AAAGACACCTACGATGATGATCTCGACAATCTGCTGGCCCAGATCGGCGACCAGTACGCAGACCTTTTTTTGG
CGGCAAAGAACCTGTCAGACGCCATTCTGCTGAGTGATATTCTGCGAGTGAACACGGAGATCACCAAAGCTC

CGCTGAGCGCTAGTATGATCAAGCGCTATGATGAGCACCACCAAGACTTGACTTTGCTGAAGGCCCTTGTCAG
ACAGCAACTGCCTGAGAAGTACAAGGAAATTTTCTTCGATCAGTCTAAAAATGGCTACGCCGGATACATTGAC
GGCGGAGCAAGCCAGGAGGAATTTTACAAATTTATTAAGCCCATCTTGGAAAAAATGGACGGCACCGAGGA
GCTGCTGGTAAAGCTTAACAGAGAAGATCTGTTGCGCAAACAGCGCACTTTCGACAATGGAAGCATCCCCCA
CCAGATTCACCTGGGCGAACTGCACGCTATCCTCAGGCGGCAAGAGGATTTCTACCCCTTTTTGAAAGATAAC
AGGGAAAAGATTGAGAAAATCCTCACATTTCGGATACCCTACTATGTAGGCCCCCTCGCCCGGGGAAATTCCA
GATTCGCGTGGATGACTCGCAAATCAGAAGAGACTATCACTCCCTGGAACTTCGAGGAAGTCGTGGATAAGG
GGGCCTCTGCCCAGTCCTTCATCGAAAGGATGACTAACTTTGATAAAAATCTGCCTAACGAAAAGGTGCTTCC
TAAACACTCTCTGCTGTACGAGTACTTCACAGTTTATAACGAGCTCACCAAGGTCAAATACGTCACAGAAGGG
ATGAGAAAGCCAGCATTCCTGTCTGGAGAGCAGAAGAAAGCTATCGTGGACCTCCTCTTCAAGACGAACCGG
AAAGTTACCGTGAAACAGCTCAAAGAAGATTATTTCAAAAAGATTGAATGTTTCGACTCTGTTGAAATCAGCG
GAGTGGAGGATCGCTTCAACGCATCCCTGGGAACGTATCACGATCTCCTGAAAATCATTAAAGACAAGGACTT
CCTGGACAATGAGGAGAACGAGGACATTCTTGAGGACATTGTCCTCACCCTTACGTTGTTTGAAGATAGGGA
GATGATTGAAGAACGCTTGAAAACTTACGCTCATCTCTTCGACGACAAAGTCATGAAACAGCTCAAGAGGCG
CCGATATACAGGATGGGGGCGGCTGTCAAGAAAACTGATCAATGGGATCCGAGACAAGCAGAGTGGAAAGA
CAATCCTGGATTTTCTTAAGTCCGATGGATTTGCCAACCGGAACTTCATGCAGTTGATCCATGATGACTCTCTC
ACCTTTAAGGAGGACATCCAGAAAGCACAAGTTTCTGGCCAGGGGGACAGTCTCCACGAGCACATCGCTAAT
CTTGCAGGTAGCCCAGCTATCAAAAAGGGAATACTGCAGACCGTTAAGGTCGTGGATGAACTCGTCAAAGTA
ATGGGAAGGCATAAGCCCGAGAATATCGTTATCGAGATGGCCCGAGAGAACCAAACTACCCAGAAGGGACA
GAAGAACAGTAGGGAAAGGATGAAGAGGATTGAAGAGGGTATAAAAGAACTGGGGTCCCAAATCCTTAAG
GAACACCCAGTTGAAAACACCCAGCTTCAGAATGAGAAGCTCTACCTGTACTACCTGCAGAACGGCAGGGAC
ATGTACGTGGATCAGGAACTGGACATCAATCGGCTCTCCGACTACGACGTGGATCATATCGTGCCCCAGTCTT
TTCTCAAAGATGATTCTATTGATAATAAAGTGTTGACAAGATCCGATAAAAATAGAGGGAAGAGTGATAACG
TCCCCTCAGAAGAAGTTGTCAAGAAAATGAAAAATTATTGGCGGCAGCTGCTGAACGCCAAACTGATCACAC
AACGGAAGTTCGATAATCTGACTAAGGCTGAACGAGGTGGCCTGTCTGAGTTGGATAAAGCCGGCTTCATCA
AAAGGCAGCTTGTTGAGACACGCCAGATCACCAAGCACGTGGCCCAAATTCTCGATTCACGCATGAACACCA
AGTACGATGAAAATGACAAACTGATTCGAGAGGTGAAAGTTATTACTCTGAAGTCTAAGCTGGTTTCAGATTT
CAGAAAGGACTTTCAGTTTTATAAGGTGAGAGAGATCAACAATTACCACCATGCGCATGATGCCTACCTGAAT
GCAGTGGTAGGCACTGCACTTATCAAAAAATATCCCAAGCTTGAATCTGAATTTGTTTACGGAGACTATAAAG
TGTACGATGTTAGGAAAATGATCGCAAAGTCTGAGCAGGAAATAGGCAAGGCCACCGCTAAGTACTTCTTTT
ACAGCAATATTATGAATTTTTTCAAGACCGAGATTACACTGGCCAATGGAGAGATTCGGAAGCGACCACTTAT
CGAAACAAACGGAGAAACAGGAGAAATCGTGTGGGACAAGGGTAGGGATTTCGCGACAGTCCGGAAGGTC
CTGTCCATGCCGCAGGTGAACATCGTTAAAAAGACCGAAGTACAGACCGGAGGCTTCTCCAAGGAAAGTATC
CTCCCGAAAAGGAACAGCGACAAGCTGATCGCACGCAAAAAAGATTGGGACCCCAAGAAATACGGCGGATT
CGATTCTCCTACAGTCGCTTACAGTGTACTGGTTGTGGCCAAAGTGGAGAAAGGGAAGTCTAAAAAACTCAA
AAGCGTCAAGGAACTGCTGGGCATCACAATCATGGAGCGATCAAGCTTCGAAAAAAACCCCATCGACTTTCTC
GAGGCGAAAGGATATAAAGAGGTCAAAAAAGACCTCATCATTAAGCTTCCCAAGTACTCTCTCTTTGAGCTTG
AAAACGGCCGGAAACGAATGCTCGCTAGTGCGGGCGAGCTGCAGAAAGGTAACGAGCTGGCACTGCCCTCT
AAATACGTTAATTTCTTGTATCTGGCCAGCCACTATGAAAAGCTCAAAGGATCTCCCGAAGATAATGAGCAGA
AGCAGCTGTTCGTGGAACAACACAAACACTACCTTGATGAGATCATCGAGCAAATAAGCGAATTCTCCAAAA
GAGTGATCCTCGCCGACGCTAACCTCGATAAGGTGCTTTCTGCTTACAATAAGCACAGGGATAAGCCCATCAG
GGAGCAGGCAGAAAACATTATCCACTTGTTTACTCTGACCAACTTGGGCGCGCCTGCAGCCTTCAAGTACTTC

GACACCACCATAGACAGAAAGCGGTACACCTCTACAAAGGAGGTCCTGGACGCCACACTGATTCATCAGTCA
ATTACGGGGCTCTATGAAACAAGAATCGACCTCTCTCAGCTCGGTGGAGACGGTTCGAGCAGGGCTGACCCC
AAGAAGAAGAGGAAGGTGGGTGGAGGAGGTACCGGCGGTGGAGGCTCAGCAGAATACGTACGAGCTCTGT
TTGACTTCAATGGGAATGACGAGGAGGATCTCCCCTTTAAGAAGGGCGATATTCTCCGCATCAGAGATAAGC
CCGAAGAACAATGGTGGAATGCCGAGGATAGCGAAGGGAAAAGGGGCATGATTCTGGTGCCATATGTGGA
GAAATATTCCGGTGACTACAAAGACCATGATGGGGATTACAAAGACCACGACATCGACTACAAAGACGACGA
CGATAAATCAGGGATGACAGACGCCGAGTACGTGCGCATTCATGAGAAACTGGATATTTACACCTTCAAGAA
GCAGTTCTTCAACAACAAGAAATCTGTGTCACACCGCTGCTACGTGCTGTTTGAGTTGAAGCGAAGGGGCGA
AAGAAGGGCTTGCTTTTGGGGCTATGCCGTCAACAAGCCCCAAAGTGGCACCGAGAGAGGAATACACGCTG
AGATATTCAGTATCCGAAAGGTGGAAGAGTATCTTCGGGATAATCCTGGGCAGTTTACGATCAACTGGTATTC
CAGCTGGAGTCCTTGCGCTGATTGTGCCGAGAAAATTCTGGAATGGTATAATCAGGAACTTCGGGGAAACGG
GCACACATTGAAAATCTGGGCCTGCAAGCTGTACTACGAGAAGAATGCCCGGAACCAGATAGGACTCTGGAA
TCTGAGGGACAATGGTGTAGGCCTGAACGTGATGGTTTCCGAGCACTATCAGTGTTGTCGGAAGATTTTCATC
CAAAGCTCTCATAACCAGCTCAATGAAAACCGCTGGTTGGAGAAAACACTGAAACGTGCGGAGAAGTGGAG
ATCCGAGCTGAGCATCATGATCCAGGTCAAGATTCTGCATACCACTAAGTCTCCAGCCGTTGGTCCCAAGAAG
AAAAGAAAAGTCGGTACCATGACCAACCTTTCCGACATCATAGAGAAGGAAACAGGCAAACAGTTGGTCATC
CAAGAGTCGATACTCATGCTTCCTGAAGAAGTTGAGGAGGTCATTGGGAATAAGCCGGAAAGTGACATTCTC
GTACACACTGCGTATGATGAGAGCACCGATGAGAACGTGATGCTGCTCACGTCAGATGCCCCAGAGTACAAA
CCCTGGGCTCTGGTGATTCAGGACTCTAATGGAGAGAACAAGATCAAGATGCTATAAGCTTCTCTAGCTAGAG
TCGATCGACAAGCTCGAGTTTCTCCATAATAATGTGTGAGTAGTTCCCAGATAAGGGAATTAGGGTTCCTATA
GGGTTTCGCTCATGTGTTGAGCATATAAGAAACCCTTAGTATGTATTTGTATTTGTAAAATACTTCTATCAATA
AAATTTCTAATTCCTAAAACCAAAATCCAGTACTAAAATCCAGAT

Blue font: CaMV 35S promoter with UBQ10 intron; black font: nCas9 (D10A, underlined); purple font: PmCDA1; orange font: UGI; red font: CaMV 35S terminator.

- **p35SI-nCas9-RT-t35S**

GAATTCCAATCCCACAAAAATCTGAGCTTAACAGCACAGTTGCTCCTCTCAGAGCAGAATCGGGTATTCAACA
CCCTCATATCAACTACTACGTTGTGTATAACGGTCCACATGCCGGTATATACGATGACTGGGGTTGTACAAAG
GCGGCAACAAACGGCGTTCCCGGAGTTGCACACAAGAAATTTGCCACTATTACAGAGGCAAGAGCAGCAGCT
GACGCGTACACAACAAGTCAGCAAACAGACAGGTTGAACTTCATCCCCAAAGGAGAAGCTCAACTCAAGCCC
AAGAGCTTTGCTAAGGCCCTAACAAGCCCACCAAAGCAAAAAGCCCACTGGCTCACGCTAGGAACCAAAAGG
CCCAGCAGTGATCCAGCCCCAAAAGAGATCTCCTTTGCCCCGGAGATTACAATGGACGATTTCCTCTATCTTTA
CGATCTAGGAAGGAAGTTCGAAGGTGAAGGTGACGACACTATGTTCACCACTGATAATGAGAAGGTTAGCCT
CTTCAATTTCAGAAAGAATGCTGACCCACAGATGGTTAGAGAGGCCTACGCAGCAAGTCTCATCAAGACGATC
TACCCGAGTAACAATCTCCAGGAGATCAAATACCTTCCCAAGAAGGTTAAAGATGCAGTCAAAAGATTCAGG
ACTAATTGCATCAAGAACACAGAGAAAGACATATTTCTCAAGATCAGAAGTACTATTCCAGTATGGACGATTC
AAGGCTTGCTTCATAAACCAAGGCAAGTAATAGAGATTGGAGTCTCTAAAAAGGTAGTTCCTACTGAATCTAA
GGCCATGCATGGAGTCTAAGATTCAAATCGAGGATCTAACAGAACTCGCCGTCAAGACTGGCGAACAGTTCA
TACAGAGTCTTTTACGACTCAATGACAAGAAGAAAATCTTCGTCAACATGGTGGAGCACGACACTCTGGTCTA
CTCCAAAAATGTCAAAGATACAGTCTCAGAAGATCAAAGGGCTATTGAGACTTTTCAACAAAGGATAATTTCG
GGAAACCTCCTCGGATTCCATTGCCCAGCTATCTGTCACTTCATCGAAAGGACAGTAGAAAAGGAAGGTGGCT

CCTACAAATGCCATCATTGCGATAAAGGAAAGGCTATCATTCAAGATCTCTCTGCCGACAGTGGTCCCAAAGA
TGGACCCCCACCCACGAGGAGCATCGTGGAAAAAGAAGAGGTTCCAACCACGTCTACAAAGCAAGTGGATTG
ATGTGACATCTCCACTGACGTAAGGGATGACGCACAATCCCACTATCCTTCGCAAGACCCTTCCTCTATATAAG
GAAGTTCATTTCATTTGGAGAGGACACGCTCGAGTATAAGGTAAATTTCTGTGTTCCTTATTCTCTCAAAATCT
TCGATTTTGTTTTCGTTCGATCCCAATTTCGTATATGTTCTTTGGTTTAGATTCTGTTAATCTTAGATCGAAGAT
GATTTTCTGGGTTTGATCGTTAGATATCATCTTAATTCTCGATTAGGGTTTCATAGATATCATCCGATTTGTTCA
AATAATTTGAGTTTTGTCGAATAATTACTCTTCGATTTGTGATTTCTATCTAGATCTGGTGTTAGTTTCTAGTTT
GTGCGATCGAATTTGTCGATTAATCTGAGTTTTTCTGATTAACAGGAGCTCATTTTTACAACAATTACCAACAA
CAACAAACAACAAACAACATTACAATTACATTTACAATTATCGATACAATGCCCAAAAAGAAAAGAAAAGTGG
ACAAGAAGTACTCCATTGGGCTCGATATCGGCACAAACAGCGTCGGCTGGGCCGTCATTACGGACGAGTACA
AGGTGCCGAGCAAAAAATTCAAAGTTCTGGGCAATACCGATCGCCACAGCATAAAGAAGAACCTCATTGGCG
CCCTCCTGTTCGACTCCGGGGAGACGGCCGAAGCCACGCGGCTCAAAAGAACAGCACGGCGCAGATATACCC
GCAGAAAGAATCGGATCTGCTACCTGCAGGAGATCTTTAGTAATGAGATGGCTAAGGTGGATGACTCTTTCTT
CCATAGGCTGGAGGAGTCCTTTTTGGTGGAGGAGGATAAAAAGCACGAGCGCCACCCAATCTTTGGCAATAT
CGTGGACGAGGTGGCGTACCATGAAAAGTACCCAACCATATATCATCTGAGGAAGAAGCTTGTAGACAGTAC
TGATAAGGCTGACTTGCGGTTGATCTATCTCGCGCTGGCGCATATGATCAAATTTCGGGGACACTTCCTCATC
GAGGGGGACCTGAACCCAGACAACAGCGATGTCGACAAACTCTTTATCCAACTGGTTCAGACTTACAATCAGC
TTTTCGAAGAGAACCCGATCAACGCATCCGGAGTTGACGCCAAAGCAATCCTGAGCGCTAGGCTGTCCAAATC
CCGGCGGCTCGAAAACCTCATCGCACAGCTCCCTGGGGAGAAGAAGAACGGCCTGTTTGGTAATCTTATCGC
CCTGTCACTCGGGCTGACCCCCAACTTTAAATCTAACTTCGACCTGGCCGAAGATGCCAAGCTTCAACTGAGC
AAAGACACCTACGATGATGATCTCGACAATCTGCTGGCCCAGATCGGCGACCAGTACGCAGACCTTTTTTTGG
CGGCAAAGAACCTGTCAGACGCCATTCTGCTGAGTGATATTCTGCGAGTGAACACGGAGATCACCAAAGCTC
CGCTGAGCGCTAGTATGATCAAGCGCTATGATGAGCACCACCAAGACTTGACTTTGCTGAAGGCCCTTGTCAG
ACAGCAACTGCCTGAGAAGTACAAGGAAATTTTCTTCGATCAGTCTAAAAATGGCTACGCCGGATACATTGAC
GGCGGAGCAAGCCAGGAGGAATTTTACAAATTTATTAAGCCCATCTTGGAAAAAATGGACGGCACCGAGGA
GCTGCTGGTAAAGCTTAACAGAGAAGATCTGTTGCGCAAACAGCGCACTTTCGACAATGGAAGCATCCCCCA
CCAGATTCACCTGGGCGAACTGCACGCTATCCTCAGGCGGCAAGAGGATTTCTACCCCTTTTTGAAAGATAAC
AGGGAAAAGATTGAGAAAATCCTCACATTTCGGATACCCTACTATGTAGGCCCCCTCGCCCGGGGAAATTCCA
GATTCGCGTGGATGACTCGCAAATCAGAAGAGACTATCACTCCCTGGAACTTCGAGGAAGTCGTGGATAAGG
GGGCCTCTGCCCAGTCCTTCATCGAAAGGATGACTAACTTTGATAAAAATCTGCCTAACGAAAAGGTGCTTCC
TAAACACTCTCTGCTGTACGAGTACTTCACAGTTTATAACGAGCTCACCAAGGTCAAATACGTCACAGAAGGG
ATGAGAAAGCCAGCATTCCTGTCTGGAGAGCAGAAGAAAGCTATCGTGGACCTCCTCTTCAAGACGAACCGG
AAAGTTACCGTGAAACAGCTCAAAGAAGATTATTTCAAAAAGATTGAATGTTTCGACTCTGTTGAAATCAGCG
GAGTGGAGGATCGCTTCAACGCATCCCTGGGAACGTATCACGATCTCCTGAAAATCATTAAAGACAAGGACTT
CCTGGACAATGAGGAGAACGAGGACATTCTTGAGGACATTGTCCTCACCCTTACGTTGTTTGAAGATAGGGA
GATGATTGAAGAACGCTTGAAAACTTACGCTCATCTCTTCGACGACAAAGTCATGAAACAGCTCAAGAGGCG
CCGATATACAGGATGGGGGCGGCTGTCAAGAAAACTGATCAATGGGATCCGAGACAAGCAGAGTGGAAAGA
CAATCCTGGATTTTCTTAAGTCCGATGGATTTGCCAACCGGAACTTCATGCAGTTGATCCATGATGACTCTCTC
ACCTTTAAGGAGGACATCCAGAAAGCACAAGTTTCTGGCCAGGGGGACAGTCTCCACGAGCACATCGCTAAT
CTTGCAGGTAGCCCAGCTATCAAAAAGGGAATACTGCAGACCGTTAAGGTCGTGGATGAACTCGTCAAAGTA
ATGGGAAGGCATAAGCCCGAGAATATCGTTATCGAGATGGCCCGAGAGAACCAAACTACCCAGAAGGGACA
GAAGAACAGTAGGGAAAGGATGAAGAGGATTGAAGAGGGTATAAAAGAACTGGGGTCCCAAATCCTTAAG

GAACACCCAGTTGAAAACACCCAGCTTCAGAATGAGAAGCTCTACCTGTACTACCTGCAGAACGGCAGGGAC
ATGTACGTGGATCAGGAACTGGACATCAATCGGCTCTCCGACTACGACGTGGATGCTATCGTGCCCCAGTCTT
TTCTCAAAGATGATTCTATTGATAATAAAGTGTTGACAAGATCCGATAAAAATAGAGGGAAGAGTGATAACG
TCCCCTCAGAAGAAGTTGTCAAGAAAATGAAAAATTATTGGCGGCAGCTGCTGAACGCCAAACTGATCACAC
AACGGAAGTTCGATAATCTGACTAAGGCTGAACGAGGTGGCCTGTCTGAGTTGGATAAAGCCGGCTTCATCA
AAAGGCAGCTTGTTGAGACACGCCAGATCACCAAGCACGTGGCCCAAATTCTCGATTCACGCATGAACACCA
AGTACGATGAAAATGACAAACTGATTCGAGAGGTGAAAGTTATTACTCTGAAGTCTAAGCTGGTTTCAGATTT
CAGAAAGGACTTTCAGTTTTATAAGGTGAGAGAGATCAACAATTACCACCATGCGCATGATGCCTACCTGAAT
GCAGTGGTAGGCACTGCACTTATCAAAAAATATCCCAAGCTTGAATCTGAATTTGTTTACGGAGACTATAAAG
TGTACGATGTTAGGAAAATGATCGCAAAGTCTGAGCAGGAAATAGGCAAGGCCACCGCTAAGTACTTCTTTT
ACAGCAATATTATGAATTTTTTCAAGACCGAGATTACACTGGCCAATGGAGAGATTCGGAAGCGACCACTTAT
CGAAACAAACGGAGAAACAGGAGAAATCGTGTGGGACAAGGGTAGGGATTTCGCGACAGTCCGGAAGGTC
CTGTCCATGCCGCAGGTGAACATCGTTAAAAAGACCGAAGTACAGACCGGAGGCTTCTCCAAGGAAAGTATC
CTCCCGAAAAGGAACAGCGACAAGCTGATCGCACGCAAAAAAGATTGGGACCCCAAGAAATACGGCGGATT
CGATTCTCCTACAGTCGCTTACAGTGTACTGGTTGTGGCCAAAGTGGAGAAAGGGAAGTCTAAAAAACTCAA
AAGCGTCAAGGAACTGCTGGGCATCACAATCATGGAGCGATCAAGCTTCGAAAAAAACCCCATCGACTTTCTC
GAGGCGAAAGGATATAAAGAGGTCAAAAAAGACCTCATCATTAAGCTTCCCAAGTACTCTCTCTTTGAGCTTG
AAAACGGCCGGAAACGAATGCTCGCTAGTGCGGGCGAGCTGCAGAAAGGTAACGAGCTGGCACTGCCCTCT
AAATACGTTAATTTCTTGTATCTGGCCAGCCACTATGAAAAGCTCAAAGGATCTCCCGAAGATAATGAGCAGA
AGCAGCTGTTCGTGGAACAACACAAACACTACCTTGATGAGATCATCGAGCAAATAAGCGAATTCTCCAAAA
GAGTGATCCTCGCCGACGCTAACCTCGATAAGGTGCTTTCTGCTTACAATAAGCACAGGGATAAGCCCATCAG
GGAGCAGGCAGAAAACATTATCCACTTGTTTACTCTGACCAACTTGGGCGCGCCTGCAGCCTTCAAGTACTTC
GACACCACCATAGACAGAAAGCGGTACACCTCTACAAAGGAGGTCCTGGACGCCACACTGATTCATCAGTCA
ATTACGGGGCTCTATGAAACAAGAATCGACCTCTCTCAGCTCGGTGGAGACGGTTCGTCTGGAGGATCTAGC
GGAGGATCCTCTGGGTCGGAAACACCAGGTACTAGCGAGTCAGCTACACCAGAGTCTAGTGGAGGCAGCAG
CGGCGGGAGTAGACACTAAATATAGAAGATGAATATCGGCTACATGAAACTTCAAAAGAGCCAGATGTTTCT
CTAGGGAGCACATGGCTATCTGATTTTCCTCAAGCCTGGGCGGAAACCGGCGGCATGGGACTGGCAGTTAGA
CAAGCTCCTCTGATTATACCACTGAAAGCAACATCTACTCCCGTTTCCATAAAACAATATCCCATGTCACAAGA
AGCCAGATTAGGAATCAAGCCTCATATACAGAGACTGTTGGACCAGGGAATACTGGTACCCTGCCAGTCCCCT
TGGAACACACCACTGCTACCCGTTAAGAAACCTGGTACTAATGATTATAGGCCTGTCCAAGATCTGAGAGAAG
TCAATAAGCGTGTGGAAGATATTCACCCTACCGTGCCAAACCCTTACAACCTCTTGTCTGGACTACCACCGTCC
CACCAATGGTACACTGTGCTTGATTTAAAGGATGCCTTTTTCTGTCTGAGACTCCATCCCACAAGTCAGCCTCT
TTTCGCCTTTGAATGGAGAGATCCAGAAATGGGGATTTCAGGACAATTGACCTGGACTAGACTCCCACAGGG
TTTCAAAAACAGTCCCACCCTGTTTAATGAGGCATTACACCGTGATTTGGCAGACTTTAGAATCCAGCACCCAG
ACTTGATTCTGCTACAGTACGTGGATGATTTACTGTTAGCCGCAACTTCTGAACTAGACTGTCAACAGGGTACT
CGAGCCCTGTTACAAACCTTGGGGAACCTTGGGTATCGGGCTTCAGCAAAGAAAGCTCAAATTTGCCAAAAA
CAAGTCAAGTATCTGGGGTATCTTCTAAAAGAGGGTCAGAGATGGCTTACTGAGGCAAGAAAAGAGACTGTG
ATGGGGCAGCCTACTCCGAAAACCCCTCGACAACTAAGGGAGTTCCTAGGGAAAGCAGGCTTCTGTCGCCTC
TTCATCCCAGGATTTGCAGAAATGGCAGCCCCCCTGTACCCTCTCACCAAACCGGGTACTCTGTTTAATTGGGG
CCCAGATCAACAAAAGGCTTATCAAGAAATTAAGCAAGCTTTGCTCACTGCCCCAGCCCTGGGTTTGCCAGAT
TTGACTAAGCCCTTTGAACTCTTTGTGGACGAGAAGCAAGGATACGCGAAAGGTGTTCTAACGCAAAAATTA
GGACCTTGGCGTAGACCAGTGGCATACCTGTCAAAAAAATTGGATCCAGTTGCAGCTGGGTGGCCTCCTTGC

CTAAGGATGGTAGCAGCCATTGCCGTACTGACAAAGGATGCCGGCAAGCTAACCATGGGACAGCCACTAGTC
ATTCTGGCCCCACATGCAGTTGAAGCACTAGTCAAGCAACCTCCCGACCGCTGGCTTTCCAATGCGCGGATGA
CTCATTATCAGGCCTTACTTTTGGATACGGACCGGGTTCAGTTCGGACCGGTGGTAGCCTTAAACCCGGCTAC
GCTGTTACCACTGCCTGAGGAAGGACTACAACACAATTGTCTTGATATTTTAGCCGAAGCACACGGAACCCGA
CCCGATCTAACGGACCAGCCGCTCCCAGATGCCGATCATACCTGGTACACTGATGGAAGCAGTTTGTTACAAG
AGGGACAGCGTAAGGCGGGAGCTGCGGTTACCACCGAGACAGAGGTAATCTGGGCTAAAGCACTGCCAGCC
GGTACATCCGCTCAGCGGGCTGAACTGATAGCACTCACCCAGGCACTAAAAATGGCAGAAGGTAAGAAGCTA
AACGTTTATACTGATAGCAGATATGCTTTTGCTACTGCTCATATCCATGGAGAAATATACAGAAGGCGTGGTT
GGCTCACATCAGAAGGCAAGGAAATCAAAAATAAAGACGAGATTTTGGCCCTACTAAAAGCCCTCTTTCTTCC
TAAAAGATTAAGCATAATTCATTGTCCAGGACATCAAAAAGGACACAGCGCCGAGGCTAGAGGTAACCGGAT
GGCTGATCAAGCTGCCCGAAAGGCAGCTATTACTGAGACTCCAGATACCTCTACACTCCTCATTGAAAATTCAT
CACCTTCTGGCGGATCAAAAAGAACCGCCGACGGTTCGGAATTCGAACCAAAGAAGAAGAGGAAAGTGTAA
GCTTCTCTAGCTAGAGTCGATCGACAAGCTCGAGTTTCTCCATAATAATGTGTGAGTAGTTCCCAGATAAGGG
AATTAGGGTTCCTATAGGGTTTCGCTCATGTGTTGAGCATATAAGAAACCCTTAGTATGTATTTGTATTTGTAA
AATACTTCTATCAATAAAATTTCTAATTCCTAAAACCAAAATCCAGTACTAAAATCCAGATC

Blue font: CaMV 35S promoter with UBQ10 intron; orange font: nCas9 (H840A, underlined); purple font: linker; dark blue font: MMLVrt; green font: SV40 NLSs; red font: CaMV 35S terminator.

- **pegRNA1_CA**

5'-
GCATGCATCGACACACACACGTTTTAGAGCTAGAAATAGCAAGTTAAAATAAGGCTAGTCCGTTATCAACTTG
AAAAAGTGGCACCGAGTCGGTGCTGTCGTGCCAGCTGCtgtcgTGTCTATCTGCCCCAGATTCTAAGCCTGTAT
GTGTGTCGATGCAT-3'
Red font sequence: gRNA; Purple sequence: 5' pgeRNA linker; Blue sequence: PBS; orange sequence: RT tem plate; green nucleotide: base substitution; black font: spCas9 scaffold.

- **sgRNA_nick for second nick on the (CA)n substrate**

GAAACCTGTCGTGCCAGCTGGTTTTAGAGCTAGAAATAGCAAGTTAAAATAAGGCTAGTCCGTTATCAACTTG
AAAAAGTGGCACCGAGTCGGTGC
Red font sequence: gRNA; black font: spCas9 scaffold

- **sgRNA_CA for base editing on the (CA)n substrate**

ACACACAGGCTTAGAATCTGGTTTTAGAGCTAGAAATAGCAAGTTAAAATAAGGCTAGTCCGTTATCAACTTG
AAAAAGTGGCACCGAGTCGGTGC
Red font sequence: gRNA; black font: spCas9 scaffold

- **pegR_HKT12**

5'-
ATCATCATGTTTTCATTTGTGTTTTAGAGCTAGAAATAGCAAGTTAAAATAAGGCTAGTCCGTTATCAACTTGA
AAAAGTGGCACCGAGTCGGTGCTTGCAgATTGTGGTTTcTTACCTACAAATGAAAACATG -3'

Red font sequence: gRNA; Blue sequence: PBS; orange sequence: RT template; green nucleotide: base substitution.

- **sgR_HKT12_2n for second nick at the SlHKT1;2**

AAACAAATCCTTGACCAAAAGTTTTAGAGCTAGAAATAGCAAGTTAAAATAAGGCTAGTCCGTTATCAACTTG AAAAAGTGGCACCGAGTCGGTGC

Red font sequence: gRNA; black font: spCas9 scaffold

- **pegR_EPSPS1**

5'-

ACAGTAACTGCTGCTGTCAAGTTTTAGAGCTAGAAATAGCAAGTTAAAATAAGGCTAGTCCGTTATCAACTTG AAAAAGTGGCACCGAGTCGGTGCAGGAAtcGCAATGCGTtctTTGACAGCAGCAGTT-3'

Red font sequence: gRNA; Blue sequence: PBS; orange sequence: RT template; green nucleotide: base substitution.

- **sgR_EPSPS1_2n for second nick at the SlEPSPS1**

AATAAATACGAATTTTGAGAGTTTTAGAGCTAGAAATAGCAAGTTAAAATAAGGCTAGTCCGTTATCAACTTG AAAAAGTGGCACCGAGTCGGTGC

Red font sequence: gRNA; black font: spCas9 scaffold

- **pegR_Or**

GAGATAATATTAGAAGTAGGGTTTTAGAGCTAGAAATAGCAAGTTAAAATAAGGCTAGTCCGTTATCAACTTG AAAAAGTGGCACCGAGTCGGTGCATCTTGTTCCTatgACTTCTAATATT

Red font sequence: gRNA; Blue sequence: PBS; orange sequence: RT template; green nucleotide: base substitution.

- **sgR_Or_2n for second nick at the SlOr**

AAAAACAAATGGCACGAAGAGTTTTAGAGCTAGAAATAGCAAGTTAAAATAAGGCTAGTCCGTTATCAACTT GAAAAAGTGGCACCGAGTCGGTGC

Red font sequence: gRNA; black font: spCas9 scaffold

- **pegR1_ MBP21**

5'-

AGCTCCTTCAACGTTCTCAAGTTTTAGAGCTAGAAATAGCAAGTTAAAATAAGGCTAGTCCGTTATCAACTTGA AAAAGTGGCACCGAGTCGGTGCTATCTTACCTTTaAGAACGTTGAAGG-3'

Red font sequence: gRNA; Blue sequence: PBS; orange sequence: RT tem plate; green nucleotide: base substitution.

- **pegR1_WH9**

  5'-
  CTTGAAGCAATCTTTAATTCGTTTTAGAGCTAGAAATAGCAAGTTAAAATAAGGCTAGTCCGTTATCAACTTGA
  AAAAGTGGCACCGAGTCGGTGCTCACCATGtCTGAATTAAAGATTGCTT-3'

  Red font sequence: gRNA; Blue sequence: PBS; orange sequence: RT tem plate; green nucleotide: base substitution.

- **pegR1_KD1**

  5'-
  TTTGACAAAGACACATTTTGTTTTAGAGCTAGAAATAGCAAGTTAAAATAAGGCTAGTCCGTTATCAACTTGA
  AAAAGTGGCACCGAGTCGGTGCACTGTAAAAAATGTTCTTTTCCAAAAATGTGTCTTTGTC-3'

  Red font sequence: gRNA; Blue sequence: PBS; orange sequence: RT tem plate; green nucleotide: base substitution.

- **pegR1_PRD**

  5'-
  AGAAAAACCAGATGCTGGAAGTTTTAGAGCTAGAAATAGCAAGTTAAAATAAGGCTAGTCCGTTATCAACTT
  GAAAAAGTGGCACCGAGTCGGTGCAAAACTAAAAGCTCATCCATTCCAGCATCTGGTTT-3'

  Red font sequence: gRNA; Blue sequence: PBS; orange sequence: RT tem plate; green nucleotide: base substitution.

- **pegR1_ALC**

  5'-
  GTTCCACCGGAAGTAAAAACGTTTTAGAGCTAGAAATAGCAAGTTAAAATAAGGCTAGTCCGTTATCAACTTG
  AAAAAGTGGCACCGAGTCGGTGCGACAAGCCGGaTTTTACTTCCGGTG-3'

  Red font sequence: gRNA; Blue sequence: PBS; orange sequence: RT tem plate; green nucleotide: base substitution.

- **pegR1_DMR6**

  5'-
  TAGAGAAGTATGCTCCTGAAGTTTTAGAGCTAGAAATAGCAAGTTAAAATAAGGCTAGTCCGTTATCAACTTG
  AAAAAGTGGCACCGAGTCGGTGCTAGAAGGCCATTaAGGAGCATACTTC-3'

  Red font sequence: gRNA; Blue sequence: PBS; orange sequence: RT template; green nucleotide: base substitution.

- **pegR1_ALS1**

5’-
CTATTACAGGTCAAGTGCCAGTTTTAGAGCTAGAAATAGCAAGTTAAAATAAGGCTAGTCCGTTATCAACTTG
AAAAAGTGGCACCGAGTCGGTGCTCATCCTCCTTGaCACTTGACCTGTA-3’

Red font sequence: gRNA; Blue sequence: PBS; orange sequence: RT template; green nucleotide: base substitution.

- **sgR1_ MBP21**

5’-
AGCTCCTTCAACGTTCTCAAGTTTTAGAGCTAGAAATAGCAAGTTAAAATAAGGCTAGTCCGTTATCAACTTGA
AAAAGTGGCACCGAGTCGGTGC-3’

Red font sequence: gRNA; black font: SpCas9 scaffold.

- **sgR1_WH9**

5’-
CTTGAAGCAATCTTTAATTCGTTTTAGAGCTAGAAATAGCAAGTTAAAATAAGGCTAGTCCGTTATCAACTTGA
AAAAGTGGCACCGAGTCGGTGC-3’

Red font sequence: gRNA; black font: SpCas9 scaffold.

- **sgR1_KD1**

5’-
TTTGACAAAGACACATTTTGTTTTAGAGCTAGAAATAGCAAGTTAAAATAAGGCTAGTCCGTTATCAACTTGA
AAAAGTGGCACCGAGTCGGTGC-3’

Red font sequence: gRNA; black font: SpCas9 scaffold.

- **sgR1_PRD**

5’-
AGAAAAACCAGATGCTGGAAGTTTTAGAGCTAGAAATAGCAAGTTAAAATAAGGCTAGTCCGTTATCAACTT
GAAAAAGTGGCACCGAGTCGGTGC-3’

Red font sequence: gRNA; black font: SpCas9 scaffold.

- **sgR1_ALC**

5’-
GTTCCACCGGAAGTAAAAACGTTTTAGAGCTAGAAATAGCAAGTTAAAATAAGGCTAGTCCGTTATCAACTTG
AAAAAGTGGCACCGAGTCGGTGC-3’

Red font sequence: gRNA; black font: SpCas9 scaffold.

- **sgR1_DMR6**

5'-
TAGAGAAGTATGCTCCTGAAGTTTTAGAGCTAGAAATAGCAAGTTAAAATAAGGCTAGTCCGTTATCAACTTG
AAAAAGTGGCACCGAGTCGGTGC-3'

Red font sequence: gRNA; black font: SpCas9 scaffold.

- **sgR1_ALS1**

5'-
CTATTACAGGTCAAGTGCCAGTTTTAGAGCTAGAAATAGCAAGTTAAAATAAGGCTAGTCCGTTATCAACTTG
AAAAAGTGGCACCGAGTCGGTGC-3'

Red font sequence: gRNA; black font: SpCas9 scaffold.

- **p35SI-Cas9-RT-t35S**

GAATTCCAATCCCACAAAAATCTGAGCTTAACAGCACAGTTGCTCCTCTCAGAGCAGAATCGGGTATTCAACA
CCCTCATATCAACTACTACGTTGTGTATAACGGTCCACATGCCGGTATATACGATGACTGGGGTTGTACAAAG
GCGGCAACAAACGGCGTTCCCGGAGTTGCACACAAGAAATTTGCCACTATTACAGAGGCAAGAGCAGCAGCT
GACGCGTACACAACAAGTCAGCAAACAGACAGGTTGAACTTCATCCCCAAAGGAGAAGCTCAACTCAAGCCC
AAGAGCTTTGCTAAGGCCCTAACAAGCCCACCAAAGCAAAAAGCCCACTGGCTCACGCTAGGAACCAAAAGG
CCCAGCAGTGATCCAGCCCCAAAAGAGATCTCCTTTGCCCCGGAGATTACAATGGACGATTTCCTCTATCTTTA
CGATCTAGGAAGGAAGTTCGAAGGTGAAGGTGACGACACTATGTTCACCACTGATAATGAGAAGGTTAGCCT
CTTCAATTTCAGAAAGAATGCTGACCCACAGATGGTTAGAGAGGCCTACGCAGCAAGTCTCATCAAGACGATC
TACCCGAGTAACAATCTCCAGGAGATCAAATACCTTCCCAAGAAGGTTAAAGATGCAGTCAAAAGATTCAGG
ACTAATTGCATCAAGAACACAGAGAAAGACATATTTCTCAAGATCAGAAGTACTATTCCAGTATGGACGATTC
AAGGCTTGCTTCATAAACCAAGGCAAGTAATAGAGATTGGAGTCTCTAAAAAGGTAGTTCCTACTGAATCTAA
GGCCATGCATGGAGTCTAAGATTCAAATCGAGGATCTAACAGAACTCGCCGTCAAGACTGGCGAACAGTTCA
TACAGAGTCTTTTACGACTCAATGACAAGAAGAAAATCTTCGTCAACATGGTGGAGCACGACACTCTGGTCTA
CTCCAAAAATGTCAAAGATACAGTCTCAGAAGATCAAAGGGCTATTGAGACTTTTCAACAAAGGATAATTTCG
GGAAACCTCCTCGGATTCCATTGCCCAGCTATCTGTCACTTCATCGAAAGGACAGTAGAAAAGGAAGGTGGCT
CCTACAAATGCCATCATTGCGATAAAGGAAAGGCTATCATTCAAGATCTCTCTGCCGACAGTGGTCCCAAAGA
TGGACCCCCACCCACGAGGAGCATCGTGGAAAAAGAAGAGGTTCCAACCACGTCTACAAAGCAAGTGGATTG
ATGTGACATCTCCACTGACGTAAGGGATGACGCACAATCCCACTATCCTTCGCAAGACCCTTCCTCTATATAAG
GAAGTTCATTTCATTTGGAGAGGACACGCTCGAGTATAAGGTAAATTTCTGTGTTCCTTATTCTCTCAAAATCT
TCGATTTTGTTTTCGTTCGATCCCAATTTCGTATATGTTCTTTGGTTTAGATTCTGTTAATCTTAGATCGAAGAT
GATTTTCTGGGTTTGATCGTTAGATATCATCTTAATTCTCGATTAGGGTTTCATAGATATCATCCGATTTGTTCA
AATAATTTGAGTTTTGTCGAATAATTACTCTTCGATTTGTGATTTCTATCTAGATCTGGTGTTAGTTTCTAGTTT
GTGCGATCGAATTTGTCGATTAATCTGAGTTTTTCTGATTAACAGGAGCTCATTTTTACAACAATTACCAACAA
CAACAAACAACAAACAACATTACAATTACATTTACAATTATCGATACAATGCCCAAAAAGAAAAGAAAAGTGG
ACAAGAAGTACTCCATTGGGCTCGATATCGGCACAAACAGCGTCGGCTGGGCCGTCATTACGGACGAGTACA
AGGTGCCGAGCAAAAAATTCAAAGTTCTGGGCAATACCGATCGCCACAGCATAAAGAAGAACCTCATTGGCG
CCCTCCTGTTCGACTCCGGGGAGACGGCCGAAGCCACGCGGCTCAAAAGAACAGCACGGCGCAGATATACCC
GCAGAAAGAATCGGATCTGCTACCTGCAGGAGATCTTTAGTAATGAGATGGCTAAGGTGGATGACTCTTTCTT

CCATAGGCTGGAGGAGTCCTTTTTGGTGGAGGAGGATAAAAAGCACGAGCGCCACCCAATCTTTGGCAATAT
CGTGGACGAGGTGGCGTACCATGAAAAGTACCCAACCATATATCATCTGAGGAAGAAGCTTGTAGACAGTAC
TGATAAGGCTGACTTGCGGTTGATCTATCTCGCGCTGGCGCATATGATCAAATTTCGGGGACACTTCCTCATC
GAGGGGGACCTGAACCCAGACAACAGCGATGTCGACAAACTCTTTATCCAACTGGTTCAGACTTACAATCAGC
TTTTCGAAGAGAACCCGATCAACGCATCCGGAGTTGACGCCAAAGCAATCCTGAGCGCTAGGCTGTCCAAATC
CCGGCGGCTCGAAAACCTCATCGCACAGCTCCCTGGGGAGAAGAAGAACGGCCTGTTTGGTAATCTTATCGC
CCTGTCACTCGGGCTGACCCCCAACTTTAAATCTAACTTCGACCTGGCCGAAGATGCCAAGCTTCAACTGAGC
AAAGACACCTACGATGATGATCTCGACAATCTGCTGGCCCAGATCGGCGACCAGTACGCAGACCTTTTTTTGG
CGGCAAAGAACCTGTCAGACGCCATTCTGCTGAGTGATATTCTGCGAGTGAACACGGAGATCACCAAAGCTC
CGCTGAGCGCTAGTATGATCAAGCGCTATGATGAGCACCACCAAGACTTGACTTTGCTGAAGGCCCTTGTCAG
ACAGCAACTGCCTGAGAAGTACAAGGAAATTTTCTTCGATCAGTCTAAAAATGGCTACGCCGGATACATTGAC
GGCGGAGCAAGCCAGGAGGAATTTTACAAATTTATTAAGCCCATCTTGGAAAAAATGGACGGCACCGAGGA
GCTGCTGGTAAAGCTTAACAGAGAAGATCTGTTGCGCAAACAGCGCACTTTCGACAATGGAAGCATCCCCCA
CCAGATTCACCTGGGCGAACTGCACGCTATCCTCAGGCGGCAAGAGGATTTCTACCCCTTTTTGAAAGATAAC
AGGGAAAAGATTGAGAAAATCCTCACATTTCGGATACCCTACTATGTAGGCCCCCTCGCCCGGGGAAATTCCA
GATTCGCGTGGATGACTCGCAAATCAGAAGAGACTATCACTCCCTGGAACTTCGAGGAAGTCGTGGATAAGG
GGGCCTCTGCCCAGTCCTTCATCGAAAGGATGACTAACTTTGATAAAAATCTGCCTAACGAAAAGGTGCTTCC
TAAACACTCTCTGCTGTACGAGTACTTCACAGTTTATAACGAGCTCACCAAGGTCAAATACGTCACAGAAGGG
ATGAGAAAGCCAGCATTCCTGTCTGGAGAGCAGAAGAAAGCTATCGTGGACCTCCTCTTCAAGACGAACCGG
AAAGTTACCGTGAAACAGCTCAAAGAAGATTATTTCAAAAAGATTGAATGTTTCGACTCTGTTGAAATCAGCG
GAGTGGAGGATCGCTTCAACGCATCCCTGGGAACGTATCACGATCTCCTGAAAATCATTAAAGACAAGGACTT
CCTGGACAATGAGGAGAACGAGGACATTCTTGAGGACATTGTCCTCACCCTTACGTTGTTTGAAGATAGGGA
GATGATTGAAGAACGCTTGAAAACTTACGCTCATCTCTTCGACGACAAAGTCATGAAACAGCTCAAGAGGCG
CCGATATACAGGATGGGGGCGGCTGTCAAGAAAACTGATCAATGGGATCCGAGACAAGCAGAGTGGAAAGA
CAATCCTGGATTTTCTTAAGTCCGATGGATTTGCCAACCGGAACTTCATGCAGTTGATCCATGATGACTCTCTC
ACCTTTAAGGAGGACATCCAGAAAGCACAAGTTTCTGGCCAGGGGGACAGTCTCCACGAGCACATCGCTAAT
CTTGCAGGTAGCCCAGCTATCAAAAAGGGAATACTGCAGACCGTTAAGGTCGTGGATGAACTCGTCAAAGTA
ATGGGAAGGCATAAGCCCGAGAATATCGTTATCGAGATGGCCCGAGAGAACCAAACTACCCAGAAGGGACA
GAAGAACAGTAGGGAAAGGATGAAGAGGATTGAAGAGGGTATAAAAGAACTGGGGTCCCAAATCCTTAAG
GAACACCCAGTTGAAAACACCCAGCTTCAGAATGAGAAGCTCTACCTGTACTACCTGCAGAACGGCAGGGAC
ATGTACGTGGATCAGGAACTGGACATCAATCGGCTCTCCGACTACGACGTGGATCATATCGTGCCCCAGTCTT
TTCTCAAAGATGATTCTATTGATAATAAAGTGTTGACAAGATCCGATAAAAATAGAGGGAAGAGTGATAACG
TCCCCTCAGAAGAAGTTGTCAAGAAAATGAAAAATTATTGGCGGCAGCTGCTGAACGCCAAACTGATCACAC
AACGGAAGTTCGATAATCTGACTAAGGCTGAACGAGGTGGCCTGTCTGAGTTGGATAAAGCCGGCTTCATCA
AAAGGCAGCTTGTTGAGACACGCCAGATCACCAAGCACGTGGCCCAAATTCTCGATTCACGCATGAACACCA
AGTACGATGAAAATGACAAACTGATTCGAGAGGTGAAAGTTATTACTCTGAAGTCTAAGCTGGTTTCAGATTT
CAGAAAGGACTTTCAGTTTTATAAGGTGAGAGAGATCAACAATTACCACCATGCGCATGATGCCTACCTGAAT
GCAGTGGTAGGCACTGCACTTATCAAAAAATATCCCAAGCTTGAATCTGAATTTGTTTACGGAGACTATAAAG
TGTACGATGTTAGGAAAATGATCGCAAAGTCTGAGCAGGAAATAGGCAAGGCCACCGCTAAGTACTTCTTTT
ACAGCAATATTATGAATTTTTTCAAGACCGAGATTACACTGGCCAATGGAGAGATTCGGAAGCGACCACTTAT
CGAAACAAACGGAGAAACAGGAGAAATCGTGTGGGACAAGGGTAGGGATTTCGCGACAGTCCGGAAGGTC
CTGTCCATGCCGCAGGTGAACATCGTTAAAAAGACCGAAGTACAGACCGGAGGCTTCTCCAAGGAAAGTATC

CTCCCGAAAAGGAACAGCGACAAGCTGATCGCACGCAAAAAAGATTGGGACCCCAAGAAATACGGCGGATT
CGATTCTCCTACAGTCGCTTACAGTGTACTGGTTGTGGCCAAAGTGGAGAAAGGGAAGTCTAAAAAACTCAA
AAGCGTCAAGGAACTGCTGGGCATCACAATCATGGAGCGATCAAGCTTCGAAAAAAACCCCATCGACTTTCTC
GAGGCGAAAGGATATAAAGAGGTCAAAAAAGACCTCATCATTAAGCTTCCCAAGTACTCTCTCTTTGAGCTTG
AAAACGGCCGGAAACGAATGCTCGCTAGTGCGGGCGAGCTGCAGAAAGGTAACGAGCTGGCACTGCCCTCT
AAATACGTTAATTTCTTGTATCTGGCCAGCCACTATGAAAAGCTCAAAGGATCTCCCGAAGATAATGAGCAGA
AGCAGCTGTTCGTGGAACAACACAAACACTACCTTGATGAGATCATCGAGCAAATAAGCGAATTCTCCAAAA
GAGTGATCCTCGCCGACGCTAACCTCGATAAGGTGCTTTCTGCTTACAATAAGCACAGGGATAAGCCCATCAG
GGAGCAGGCAGAAAACATTATCCACTTGTTTACTCTGACCAACTTGGGCGCGCCTGCAGCCTTCAAGTACTTC
GACACCACCATAGACAGAAAGCGGTACACCTCTACAAAGGAGGTCCTGGACGCCACACTGATTCATCAGTCA
ATTACGGGGCTCTATGAAACAAGAATCGACCTCTCTCAGCTCGGTGGAGACGGTTCGTCTGGAGGATCTAGC
GGAGGATCCTCTGGGTCGGAAACACCAGGTACTAGCGAGTCAGCTACACCAGAGTCTAGTGGAGGCAGCAG
CGGCGGGAGTAGACACTAAATATAGAAGATGAATATCGGCTACATGAAACTTCAAAAGAGCCAGATGTTTCT
CTAGGGAGCACATGGCTATCTGATTTTCCTCAAGCCTGGGCGGAAACCGGCGGCATGGGACTGGCAGTTAGA
CAAGCTCCTCTGATTATACCACTGAAAGCAACATCTACTCCCGTTTCCATAAAACAATATCCCATGTCACAAGA
AGCCAGATTAGGAATCAAGCCTCATATACAGAGACTGTTGGACCAGGGAATACTGGTACCCTGCCAGTCCCCT
TGGAACACACCACTGCTACCCGTTAAGAAACCTGGTACTAATGATTATAGGCCTGTCCAAGATCTGAGAGAAG
TCAATAAGCGTGTGGAAGATATTCACCCTACCGTGCCAAACCCTTACAACCTCTTGTCTGGACTACCACCGTCC
CACCAATGGTACACTGTGCTTGATTTAAAGGATGCCTTTTTCTGTCTGAGACTCCATCCCACAAGTCAGCCTCT
TTTCGCCTTTGAATGGAGAGATCCAGAAATGGGGATTTCAGGACAATTGACCTGGACTAGACTCCCACAGGG
TTTCAAAAACAGTCCCACCCTGTTTAATGAGGCATTACACCGTGATTTGGCAGACTTTAGAATCCAGCACCCAG
ACTTGATTCTGCTACAGTACGTGGATGATTTACTGTTAGCCGCAACTTCTGAACTAGACTGTCAACAGGGTACT
CGAGCCCTGTTACAAACCTTGGGGAACCTTGGGTATCGGGCTTCAGCAAAGAAAGCTCAAATTTGCCAAAAA
CAAGTCAAGTATCTGGGGTATCTTCTAAAAGAGGGTCAGAGATGGCTTACTGAGGCAAGAAAAGAGACTGTG
ATGGGGCAGCCTACTCCGAAAACCCCTCGACAACTAAGGGAGTTCCTAGGGAAAGCAGGCTTCTGTCGCCTC
TTCATCCCAGGATTTGCAGAAATGGCAGCCCCCCTGTACCCTCTCACCAAACCGGGTACTCTGTTTAATTGGGG
CCCAGATCAACAAAAGGCTTATCAAGAAATTAAGCAAGCTTTGCTCACTGCCCCAGCCCTGGGTTTGCCAGAT
TTGACTAAGCCCTTTGAACTCTTTGTGGACGAGAAGCAAGGATACGCGAAAGGTGTTCTAACGCAAAAATTA
GGACCTTGGCGTAGACCAGTGGCATACCTGTCAAAAAAATTGGATCCAGTTGCAGCTGGGTGGCCTCCTTGC
CTAAGGATGGTAGCAGCCATTGCCGTACTGACAAAGGATGCCGGCAAGCTAACCATGGGACAGCCACTAGTC
ATTCTGGCCCCACATGCAGTTGAAGCACTAGTCAAGCAACCTCCCGACCGCTGGCTTTCCAATGCGCGGATGA
CTCATTATCAGGCCTTACTTTTGGATACGGACCGGGTTCAGTTCGGACCGGTGGTAGCCTTAAACCCGGCTAC
GCTGTTACCACTGCCTGAGGAAGGACTACAACACAATTGTCTTGATATTTTAGCCGAAGCACACGGAACCCGA
CCCGATCTAACGGACCAGCCGCTCCCAGATGCCGATCATACCTGGTACACTGATGGAAGCAGTTTGTTACAAG
AGGGACAGCGTAAGGCGGGAGCTGCGGTTACCACCGAGACAGAGGTAATCTGGGCTAAAGCACTGCCAGCC
GGTACATCCGCTCAGCGGGCTGAACTGATAGCACTCACCCAGGCACTAAAAATGGCAGAAGGTAAGAAGCTA
AACGTTTATACTGATAGCAGATATGCTTTTGCTACTGCTCATATCCATGGAGAAATATACAGAAGGCGTGGTT
GGCTCACATCAGAAGGCAAGGAAATCAAAAATAAAGACGAGATTTTGGCCCTACTAAAAGCCCTCTTTCTTCC
TAAAAGATTAAGCATAATTCATTGTCCAGGACATCAAAAAGGACACAGCGCCGAGGCTAGAGGTAACCGGAT
GGCTGATCAAGCTGCCCGAAAGGCAGCTATTACTGAGACTCCAGATACCTCTACACTCCTCATTGAAAATTCAT
CACCTTCTGGCGGATCAAAAAGAACCGCCGACGGTTCGGAATTCGAACCAAAGAAGAAGAGGAAAGTGTAA
GCTTCTCTAGCTAGAGTCGATCGACAAGCTCGAGTTTCTCCATAATAATGTGTGAGTAGTTCCCAGATAAGGG

AATTAGGGTTCCTATAGGGTTTCGCTCATGTGTTGAGCATATAAGAAACCCTTAGTATGTATTTGTATTTGTAA
AATACTTCTATCAATAAAATTTCTAATTCCTAAAACCAAAATCCAGTACTAAAATCCAGATC

Blue font: CaMV 35S promoter with UBQ10 intron; orange font: full function Cas9 (H840, underlined); purple font: linker; dark blue font: MMLVrt; green font: SV40 NLSs; red font: CaMV 35S terminator.

- **p35SI-Cas9-t35S**

GAATTCCAATCCCACAAAAATCTGAGCTTAACAGCACAGTTGCTCCTCTCAGAGCAGAATCGGGTATTCAACA
CCCTCATATCAACTACTACGTTGTGTATAACGGTCCACATGCCGGTATATACGATGACTGGGGTTGTACAAAG
GCGGCAACAAACGGCGTTCCCGGAGTTGCACACAAGAAATTTGCCACTATTACAGAGGCAAGAGCAGCAGCT
GACGCGTACACAACAAGTCAGCAAACAGACAGGTTGAACTTCATCCCCAAAGGAGAAGCTCAACTCAAGCCC
AAGAGCTTTGCTAAGGCCCTAACAAGCCCACCAAAGCAAAAAGCCCACTGGCTCACGCTAGGAACCAAAAGG
CCCAGCAGTGATCCAGCCCCAAAAGAGATCTCCTTTGCCCCGGAGATTACAATGGACGATTTCCTCTATCTTTA
CGATCTAGGAAGGAAGTTCGAAGGTGAAGGTGACGACACTATGTTCACCACTGATAATGAGAAGGTTAGCCT
CTTCAATTTCAGAAAGAATGCTGACCCACAGATGGTTAGAGAGGCCTACGCAGCAAGTCTCATCAAGACGATC
TACCCGAGTAACAATCTCCAGGAGATCAAATACCTTCCCAAGAAGGTTAAAGATGCAGTCAAAAGATTCAGG
ACTAATTGCATCAAGAACACAGAGAAAGACATATTTCTCAAGATCAGAAGTACTATTCCAGTATGGACGATTC
AAGGCTTGCTTCATAAACCAAGGCAAGTAATAGAGATTGGAGTCTCTAAAAAGGTAGTTCCTACTGAATCTAA
GGCCATGCATGGAGTCTAAGATTCAAATCGAGGATCTAACAGAACTCGCCGTCAAGACTGGCGAACAGTTCA
TACAGAGTCTTTTACGACTCAATGACAAGAAGAAAATCTTCGTCAACATGGTGGAGCACGACACTCTGGTCTA
CTCCAAAAATGTCAAAGATACAGTCTCAGAAGATCAAAGGGCTATTGAGACTTTTCAACAAAGGATAATTTCG
GGAAACCTCCTCGGATTCCATTGCCCAGCTATCTGTCACTTCATCGAAAGGACAGTAGAAAAGGAAGGTGGCT
CCTACAAATGCCATCATTGCGATAAAGGAAAGGCTATCATTCAAGATCTCTCTGCCGACAGTGGTCCCAAAGA
TGGACCCCCACCCACGAGGAGCATCGTGGAAAAAGAAGAGGTTCCAACCACGTCTACAAAGCAAGTGGATTG
ATGTGACATCTCCACTGACGTAAGGGATGACGCACAATCCCACTATCCTTCGCAAGACCCTTCCTCTATATAAG
GAAGTTCATTTCATTTGGAGAGGACACGCTCGAGTATAAGGTAAATTTCTGTGTTCCTTATTCTCTCAAAATCT
TCGATTTTGTTTTCGTTCGATCCCAATTTCGTATATGTTCTTTGGTTTAGATTCTGTTAATCTTAGATCGAAGAT
GATTTTCTGGGTTTGATCGTTAGATATCATCTTAATTCTCGATTAGGGTTTCATAGATATCATCCGATTTGTTCA
AATAATTTGAGTTTTGTCGAATAATTACTCTTCGATTTGTGATTTCTATCTAGATCTGGTGTTAGTTTCTAGTTT
GTGCGATCGAATTTGTCGATTAATCTGAGTTTTTCTGATTAACAGGAGCTCATTTTTACAACAATTACCAACAA
CAACAAACAACAAACAACATTACAATTACATTTACAATTATCGATACAATGCCCAAAAAGAAAAGAAAAGTGG
ACAAGAAGTACTCCATTGGGCTCGATATCGGCACAAACAGCGTCGGCTGGGCCGTCATTACGGACGAGTACA
AGGTGCCGAGCAAAAAATTCAAAGTTCTGGGCAATACCGATCGCCACAGCATAAAGAAGAACCTCATTGGCG
CCCTCCTGTTCGACTCCGGGGAGACGGCCGAAGCCACGCGGCTCAAAAGAACAGCACGGCGCAGATATACCC
GCAGAAAGAATCGGATCTGCTACCTGCAGGAGATCTTTAGTAATGAGATGGCTAAGGTGGATGACTCTTTCTT
CCATAGGCTGGAGGAGTCCTTTTTGGTGGAGGAGGATAAAAAGCACGAGCGCCACCCAATCTTTGGCAATAT
CGTGGACGAGGTGGCGTACCATGAAAAGTACCCAACCATATATCATCTGAGGAAGAAGCTTGTAGACAGTAC
TGATAAGGCTGACTTGCGGTTGATCTATCTCGCGCTGGCGCATATGATCAAATTTCGGGGACACTTCCTCATC
GAGGGGGACCTGAACCCAGACAACAGCGATGTCGACAAACTCTTTATCCAACTGGTTCAGACTTACAATCAGC
TTTTCGAAGAGAACCCGATCAACGCATCCGGAGTTGACGCCAAAGCAATCCTGAGCGCTAGGCTGTCCAAATC
CCGGCGGCTCGAAAACCTCATCGCACAGCTCCCTGGGGAGAAGAAGAACGGCCTGTTTGGTAATCTTATCGC

CCTGTCACTCGGGCTGACCCCCAACTTTAAATCTAACTTCGACCTGGCCGAAGATGCCAAGCTTCAACTGAGC
AAAGACACCTACGATGATGATCTCGACAATCTGCTGGCCCAGATCGGCGACCAGTACGCAGACCTTTTTTTGG
CGGCAAAGAACCTGTCAGACGCCATTCTGCTGAGTGATATTCTGCGAGTGAACACGGAGATCACCAAAGCTC
CGCTGAGCGCTAGTATGATCAAGCGCTATGATGAGCACCACCAAGACTTGACTTTGCTGAAGGCCCTTGTCAG
ACAGCAACTGCCTGAGAAGTACAAGGAAATTTTCTTCGATCAGTCTAAAAATGGCTACGCCGGATACATTGAC
GGCGGAGCAAGCCAGGAGGAATTTTACAAATTTATTAAGCCCATCTTGGAAAAAATGGACGGCACCGAGGA
GCTGCTGGTAAAGCTTAACAGAGAAGATCTGTTGCGCAAACAGCGCACTTTCGACAATGGAAGCATCCCCCA
CCAGATTCACCTGGGCGAACTGCACGCTATCCTCAGGCGGCAAGAGGATTTCTACCCCTTTTTGAAAGATAAC
AGGGAAAAGATTGAGAAAATCCTCACATTTCGGATACCCTACTATGTAGGCCCCCTCGCCCGGGGAAATTCCA
GATTCGCGTGGATGACTCGCAAATCAGAAGAGACTATCACTCCCTGGAACTTCGAGGAAGTCGTGGATAAGG
GGGCCTCTGCCCAGTCCTTCATCGAAAGGATGACTAACTTTGATAAAAATCTGCCTAACGAAAAGGTGCTTCC
TAAACACTCTCTGCTGTACGAGTACTTCACAGTTTATAACGAGCTCACCAAGGTCAAATACGTCACAGAAGGG
ATGAGAAAGCCAGCATTCCTGTCTGGAGAGCAGAAGAAAGCTATCGTGGACCTCCTCTTCAAGACGAACCGG
AAAGTTACCGTGAAACAGCTCAAAGAAGATTATTTCAAAAAGATTGAATGTTTCGACTCTGTTGAAATCAGCG
GAGTGGAGGATCGCTTCAACGCATCCCTGGGAACGTATCACGATCTCCTGAAAATCATTAAAGACAAGGACTT
CCTGGACAATGAGGAGAACGAGGACATTCTTGAGGACATTGTCCTCACCCTTACGTTGTTTGAAGATAGGGA
GATGATTGAAGAACGCTTGAAAACTTACGCTCATCTCTTCGACGACAAAGTCATGAAACAGCTCAAGAGGCG
CCGATATACAGGATGGGGGCGGCTGTCAAGAAAACTGATCAATGGGATCCGAGACAAGCAGAGTGGAAAGA
CAATCCTGGATTTTCTTAAGTCCGATGGATTTGCCAACCGGAACTTCATGCAGTTGATCCATGATGACTCTCTC
ACCTTTAAGGAGGACATCCAGAAAGCACAAGTTTCTGGCCAGGGGGACAGTCTCCACGAGCACATCGCTAAT
CTTGCAGGTAGCCCAGCTATCAAAAAGGGAATACTGCAGACCGTTAAGGTCGTGGATGAACTCGTCAAAGTA
ATGGGAAGGCATAAGCCCGAGAATATCGTTATCGAGATGGCCCGAGAGAACCAAACTACCCAGAAGGGACA
GAAGAACAGTAGGGAAAGGATGAAGAGGATTGAAGAGGGTATAAAAGAACTGGGGTCCCAAATCCTTAAG
GAACACCCAGTTGAAAACACCCAGCTTCAGAATGAGAAGCTCTACCTGTACTACCTGCAGAACGGCAGGGAC
ATGTACGTGGATCAGGAACTGGACATCAATCGGCTCTCCGACTACGACGTGGATCATATCGTGCCCCAGTCTT
TTCTCAAAGATGATTCTATTGATAATAAAGTGTTGACAAGATCCGATAAAAATAGAGGGAAGAGTGATAACG
TCCCCTCAGAAGAAGTTGTCAAGAAAATGAAAAATTATTGGCGGCAGCTGCTGAACGCCAAACTGATCACAC
AACGGAAGTTCGATAATCTGACTAAGGCTGAACGAGGTGGCCTGTCTGAGTTGGATAAAGCCGGCTTCATCA
AAAGGCAGCTTGTTGAGACACGCCAGATCACCAAGCACGTGGCCCAAATTCTCGATTCACGCATGAACACCA
AGTACGATGAAAATGACAAACTGATTCGAGAGGTGAAAGTTATTACTCTGAAGTCTAAGCTGGTTTCAGATTT
CAGAAAGGACTTTCAGTTTTATAAGGTGAGAGAGATCAACAATTACCACCATGCGCATGATGCCTACCTGAAT
GCAGTGGTAGGCACTGCACTTATCAAAAAATATCCCAAGCTTGAATCTGAATTTGTTTACGGAGACTATAAAG
TGTACGATGTTAGGAAAATGATCGCAAAGTCTGAGCAGGAAATAGGCAAGGCCACCGCTAAGTACTTCTTTT
ACAGCAATATTATGAATTTTTTCAAGACCGAGATTACACTGGCCAATGGAGAGATTCGGAAGCGACCACTTAT
CGAAACAAACGGAGAAACAGGAGAAATCGTGTGGGACAAGGGTAGGGATTTCGCGACAGTCCGGAAGGTC
CTGTCCATGCCGCAGGTGAACATCGTTAAAAAGACCGAAGTACAGACCGGAGGCTTCTCCAAGGAAAGTATC
CTCCCGAAAAGGAACAGCGACAAGCTGATCGCACGCAAAAAAGATTGGGACCCCAAGAAATACGGCGGATT
CGATTCTCCTACAGTCGCTTACAGTGTACTGGTTGTGGCCAAAGTGGAGAAAGGGAAGTCTAAAAAACTCAA
AAGCGTCAAGGAACTGCTGGGCATCACAATCATGGAGCGATCAAGCTTCGAAAAAAACCCCATCGACTTTCTC
GAGGCGAAAGGATATAAAGAGGTCAAAAAAGACCTCATCATTAAGCTTCCCAAGTACTCTCTCTTTGAGCTTG
AAAACGGCCGGAAACGAATGCTCGCTAGTGCGGGCGAGCTGCAGAAAGGTAACGAGCTGGCACTGCCCTCT
AAATACGTTAATTTCTTGTATCTGGCCAGCCACTATGAAAAGCTCAAAGGATCTCCCGAAGATAATGAGCAGA

AGCAGCTGTTCGTGGAACAACACAAACACTACCTTGATGAGATCATCGAGCAAATAAGCGAATTCTCCAAAA
GAGTGATCCTCGCCGACGCTAACCTCGATAAGGTGCTTTCTGCTTACAATAAGCACAGGGATAAGCCCATCAG
GGAGCAGGCAGAAAACATTATCCACTTGTTTACTCTGACCAACTTGGGCGCGCCTGCAGCCTTCAAGTACTTC
GACACCACCATAGACAGAAAGCGGTACACCTCTACAAAGGAGGTCCTGGACGCCACACTGATTCATCAGTCA
ATTACGGGGCTCTATGAAACAAGAATCGACCTCTCTCAGCTCGGTGGAGACTCTGGCGGATCAAAAAGAACC
GCCGACGGTTCGGAATTCGAACCAAAGAAGAAGAGGAAAGTGTAAGCTTCTCTAGCTAGAGTCGATCGACAA
GCTCGAGTTTCTCCATAATAATGTGTGAGTAGTTCCCAGATAAGGGAATTAGGGTTCCTATAGGGTTTCGCTC
ATGTGTTGAGCATATAAGAAACCCTTAGTATGTATTTGTATTTGTAAAATACTTCTATCAATAAAATTTCTAATT
CCTAAAACCAAAATCCAGTACTAAAATCCAGATC

Blue font: CaMV 35S promoter with UBQ10 intron; orange font: full function Cas9 (H840, underlined); green font: SV40 NLSs; red font: CaMV 35S terminator.

- **p35SI-nCas9-PPE-t35S expression cassette**:

GGAGGAATTCCAATCCCACAAAAATCTGAGCTTAACAGCACAGTTGCTCCTCTCAGAGCAGAATCGGGTATTC
AACACCCTCATATCAACTACTACGTTGTGTATAACGGTCCACATGCCGGTATATACGATGACTGGGGTTGTAC
AAAGGCGGCAACAAACGGCGTTCCCGGAGTTGCACACAAGAAATTTGCCACTATTACAGAGGCAAGAGCAG
CAGCTGACGCGTACACAACAAGTCAGCAAACAGACAGGTTGAACTTCATCCCCAAAGGAGAAGCTCAACTCA
AGCCCAAGAGCTTTGCTAAGGCCCTAACAAGCCCACCAAAGCAAAAAGCCCACTGGCTCACGCTAGGAACCA
AAAGGCCCAGCAGTGATCCAGCCCCAAAAGAGATCTCCTTTGCCCCGGAGATTACAATGGACGATTTCCTCTA
TCTTTACGATCTAGGAAGGAAGTTCGAAGGTGAAGGTGACGACACTATGTTCACCACTGATAATGAGAAGGT
TAGCCTCTTCAATTTCAGAAAGAATGCTGACCCACAGATGGTTAGAGAGGCCTACGCAGCAAGTCTCATCAAG
ACGATCTACCCGAGTAACAATCTCCAGGAGATCAAATACCTTCCCAAGAAGGTTAAAGATGCAGTCAAAAGAT
TCAGGACTAATTGCATCAAGAACACAGAGAAAGACATATTTCTCAAGATCAGAAGTACTATTCCAGTATGGAC
GATTCAAGGCTTGCTTCATAAACCAAGGCAAGTAATAGAGATTGGAGTCTCTAAAAAGGTAGTTCCTACTGAA
TCTAAGGCCATGCATGGAGTCTAAGATTCAAATCGAGGATCTAACAGAACTCGCCGTCAAGACTGGCGAACA
GTTCATACAGAGTCTTTTACGACTCAATGACAAGAAGAAAATCTTCGTCAACATGGTGGAGCACGACACTCTG
GTCTACTCCAAAAATGTCAAAGATACAGTCTCAGAAGATCAAAGGGCTATTGAGACTTTTCAACAAAGGATAA
TTTCGGGAAACCTCCTCGGATTCCATTGCCCAGCTATCTGTCACTTCATCGAAAGGACAGTAGAAAAGGAAGG
TGGCTCCTACAAATGCCATCATTGCGATAAAGGAAAGGCTATCATTCAAGATCTCTCTGCCGACAGTGGTCCC
AAAGATGGACCCCCACCCACGAGGAGCATCGTGGAAAAAGAAGAGGTTCCAACCACGTCTACAAAGCAAGT
GGATTGATGTGACATCTCCACTGACGTAAGGGATGACGCACAATCCCACTATCCTTCGCAAGACCCTTCCTCTA
TATAAGGAAGTTCATTTCATTTGGAGAGGACACGCTCGAGTATAAGGTAAATTTCTGTGTTCCTTATTCTCTCA
AAATCTTCGATTTTGTTTTCGTTCGATCCCAATTTCGTATATGTTCTTTGGTTTAGATTCTGTTAATCTTAGATCG
AAGATGATTTTCTGGGTTTGATCGTTAGATATCATCTTAATTCTCGATTAGGGTTTCATAGATATCATCCGATTT
GTTCAAATAATTTGAGTTTTGTCGAATAATTACTCTTCGATTTGTGATTTCTATCTAGATCTGGTGTTAGTTTCT
AGTTTGTGCGATCGAATTTGTCGATTAATCTGAGTTTTTCTGATTAACAGGAGCTCATTTTTACAACAATTACCA
ACAACAACAAACAACAAACAACATTACAATTACATTTACAATTATCGATACAATGCCTAAGAAAAAGAGAAAA
GTAAGACGATCCTCGACTTCCTGAAGAGCGATGGCTTCGCGAACCGCAATTTCATGCAGCTGATTCACGATGA
CAGCCTCACATTCAAGGAGGATATCCAGAAGGCTCAGGTGAGCGGCCAGGGGGACTCGCTGCACGAGCATA
TCGCGAACCTCGCTGGCTCGCCAGCTATCAAGAAGGGGATTCTGCAGACCGTGAAGGTTGTGGACGAGCTGG
TGAAGGTCATGGGCAGGCACAAGCCTGAGAACATCGTCATTGAGATGGCCCGGGAGAATCAGACCACGCAG

AAGGGCCAGAAGAACTCACGCGAGAGGATGAAGAGGATCGAGGAGGGCATTAAGGAGCTGGGGTCCCAGA
TCCTCAAGGAGCACCCGGTGGAGAACACGCAGCTGCAGAATGAGAAGCTCTACCTGTACTACCTCCAGAATG
GCCGCGATATGTATGTGGACCAGGAGCTGGATATTAACAGGCTCAGCGATTACGACGTCGATGCCATCGTTC
CACAGTCATTCCTGAAGGATGACTCCATTGACAACAAGGTCCTCACCAGGTCGGACAAGAACCGGGGCAAGT
CTGATAATGTTCCTTCAGAGGAGGTCGTTAAGAAGATGAAGAACTACTGGCGCCAGCTCCTGAATGCCAAGC
TGATCACGCAGCGGAAGTTCGATAACCTCACAAAGGCTGAGAGGGGCGGGCTCTCTGAGCTGGACAAGGCG
GGCTTCATCAAGAGGCAGCTGGTCGAGACACGGCAGATCACTAAGCACGTTGCGCAGATTCTCGACTCACGG
ATGAACACTAAGTACGATGAGAATGACAAGCTGATCCGCGAGGTGAAGGTCATCACCCTGAAGTCAAAGCTC
GTCTCCGACTTCAGGAAGGATTTCCAGTTCTACAAGGTTCGGGAGATCAACAATTACCACCATGCCCATGACG
CGTACCTGAACGCGGTGGTCGGCACAGCTCTGATCAAGAAGTACCCAAAGCTCGAGAGCGAGTTCGTGTACG
GGGACTACAAGGTTTACGATGTGAGGAAGATGATCGCCAAGTCGGAGCAGGAGATTGGCAAGGCTACCGCC
AAGTACTTCTTCTACTCTAACATTATGAATTTCTTCAAGACAGAGATCACTCTGGCCAATGGCGAGATCCGGAA
GCGCCCCCTCATCGAGACGAACGGCGAGACGGGGGAGATCGTGTGGGACAAGGGCAGGGATTTCGCGACC
GTCAGGAAGGTTCTCTCCATGCCACAAGTGAATATCGTCAAAAAGACAGAGGTCCAGACTGGCGGGTTCTCT
AAGGAGTCAATTCTGCCTAAGCGGAACAGCGACAAGCTCATCGCCCGCAAGAAGGACTGGGATCCGAAGAA
GTACGGCGGGTTCGACAGCCCCACTGTGGCCTACTCGGTCCTGGTTGTGGCGAAGGTTGAGAAGGGCAAGTC
CAAGAAGCTCAAGAGCGTGAAGGAGCTGCTGGGGATCACGATTATGGAGCGCTCCAGCTTCGAGAAGAACC
CGATCGATTTCCTGGAGGCGAAGGGCTACAAGGAGGTGAAGAAGGACCTGATCATTAAGCTCCCCAAGTACT
CACTCTTCGAGCTGGAGAACGGCAGGAAGCGGATGCTGGCTTCCGCTGGCGAGCTGCAGAAGGGGAACGAG
CTGGCTCTGCCGTCCAAGTATGTGAACTTCCTCTACCTGGCCTCCCACTACGAGAAGCTCAAGGGCAGCCCCG
AGGACAACGAGCAGAAGCAGCTGTTCGTCGAGCAGCACAAGCATTACCTCGACGAGATCATTGAGCAGATTT
CCGAGTTCTCCAAGCGCGTGATCCTGGCCGACGCGAATCTGGATAAGGTCCTCTCCGCGTACAACAAGCACC
GCGACAAGCCAATCAGGGAGCAGGCTGAGAATATCATTCATCTCTTCACCCTGACGAACCTCGGCGCCCCTGC
TGCTTTCAAGTACTTCGACACAACTATCGATCGCAAGAGGTACACAAGCACTAAGGAGGTCCTGGACGCGAC
CCTCATCCACCAGTCGATTACCGGCCTCTACGAGACGCGCATCGACCTGTCTCAGCTCGGGGGCGACGAATTC
CCAAAGAAGAAGCGGAAGGTGGAGCTCAGCGGAGGATCTTCCGGAGGATCTAGCGGCTCCGAGACACCAG
GAACATCCGAAAGCGCTACACCAGAATCTAGCGGAGGCTCTTCCGGAGGATCTAGGCCTACCCTCAACATCG
AGGATGAGTATCGCCTCCACGAAACCTCCAAAGAACCGGACGTGTCCCTCGGCAGCACATGGCTCAGCGACT
TCCCACAAGCGTGGGCCGAAACCGGCGGCATGGGCCTCGCCGTCCGCCAAGCCCCACTCATTATCCCGCTGA
AGGCGACCTCCACACCGGTGTCCATCAAGCAGTACCCGATGAGCCAAGAGGCGAGGCTCGGGATTAAGCCG
CACATTCAGCGCCTCCTCGATCAAGGCATTCTCGTGCCGTGCCAATCCCCGTGGAATACACCACTCCTCCCGGT
CAAAAAGCCGGGCACCAACGACTATCGCCCGGTCCAAGATCTCCGCGAGGTCAACAAGCGCGTGGAAGATAT
CCACCCGACCGTCCCGAACCCGTATAATCTGCTCTCCGGGCTCCCACCATCCCACCAGTGGTATACAGTGCTG
GACCTCAAAGACGCCTTCTTCTGTCTCCGCCTCCACCCAACAAGCCAGCCGCTCTTCGCCTTCGAGTGGCGCGA
CCCGGAGATGGGCATCTCCGGCCAACTGACATGGACACGCCTCCCGCAAGGCTTCAAGAACAGCCCGACACT
CTTCAACGAGGCGCTCCATAGGGACCTCGCGGATTTTCGCATCCAGCATCCGGACCTCATCCTCCTCCAGTATG
TGGATGATCTCCTCCTCGCCGCGACCTCCGAGCTGGATTGTCAACAAGGCACACGCGCGCTCCTCCAAACACT
CGGGAACCTCGGCTATCGCGCGTCCGCGAAAAAGGCCCAAATCTGCCAGAAGCAAGTGAAGTACCTCGGGTA
TCTGCTCAAGGAAGGCCAACGCTGGCTCACCGAAGCGCGCAAAGAAACAGTGATGGGGCAACCGACACCGA
AAACACCACGCCAGCTGCGCGAGTTTCTCGGCAAAGCCGGCTTCTGTCGCCTCTTCATCCCGGGCTTTGCCGA
GATGGCCGCGCCACTCTACCCACTCACCAAGCCGGGCACACTGTTTAACTGGGGGCCGGATCAGCAGAAAGC
CTACCAAGAGATCAAACAAGCGCTCCTCACCGCCCCAGCGCTCGGGCTCCCAGATCTCACAAAGCCGTTCGAG

CTGTTCGTCGATGAGAAGCAAGGCTACGCGAAGGGCGTGCTCACACAGAAGCTCGGCCCGTGGAGGAGGCC
AGTGGCCTATCTCTCCAAAAAACTCGATCCAGTGGCCGCCGGCTGGCCACCGTGTCTGCGCATGGTCGCCGCG
ATTGCCGTGCTCACAAAGGATGCCGGCAAACTCACAATGGGCCAGCCGCTGGTGATCCTCGCGCCACATGCC
GTGGAAGCCCTCGTCAAACAGCCGCCGGATAGGTGGCTCTCCAATGCGCGCATGACCCATTACCAAGCGCTC
CTCCTCGACACCGATCGCGTCCAGTTCGGCCCAGTGGTCGCCCTCAATCCGGCGACACTGCTGCCACTCCCAG
AGGAGGGCCTCCAACACAACTGTCTGGATATTCTCGCGGAAGCGCATGGCACAAGGCCAGACCTCACAGATC
AACCGCTCCCGGATGCGGATCACACATGGTATACCGACGGCTCCTCTCTGCTCCAAGAGGGCCAAAGGAAAG
CCGGCGCCGCGGTGACCACAGAAACAGAAGTGATCTGGGCCAAGGCCCTCCCAGCCGGCACATCCGCGCAA
AGGGCGGAACTCATCGCGCTCACACAAGCCCTCAAGATGGCCGAGGGCAAGAAGCTCAACGTCTACACAGAC
TCCCGCTATGCCTTCGCCACCGCCCACATTCACGGCGAAATCTATAGGAGGCGCGGCTGGCTCACAAGCGAG
GGGAAGGAGATCAAGAACAAGGATGAGATCCTCGCGCTGCTCAAGGCCCTCTTTCTCCCGAAGCGCCTCTCC
ATCATCCACTGTCCGGGCCACCAAAAGGGGCACTCCGCGGAAGCGAGGGGCAATAGGATGGCCGATCAAGC
CGCGCGCAAAGCCGCGATTACCGAAACCCCAGACACATCCACCCTCCTCATCGAAAACTCCTCCCCAAGCGGC
GGCAGCCCGAAGAAGAAAAGGAAGGTGTAAGCTTCTCTAGCTAGAGTCGATCGACAAGCTCGAGTTTCTCCA
TAATAATGTGTGAGTAGTTCCCAGATAAGGGAATTAGGGTTCCTATAGGGTTTCGCTCATGTGTTGAGCATAT
AAGAAACCCTTAGTATGTATTTGTATTTGTAAAATACTTCTATCAATAAAATTTCTAATTCCTAAAACCAAAATC
CAGTACTAAAATCCAGAT

Blue font: CaMV 35S promoter with UBQ10 intron; orange font: nCas9 (H840A, underlined); purple font: linker; dark blue font: MMLVrt; green font: SV40 NLSs; red font: CaMV 35S terminator.

- **Plant selection marker**: pNOS-NptII-tOCS cloned from pICSL11024 (pICH47732::NOSp-NPTII-OCST) (Addgene Plasmid #51144).
